# Opposing Functions of Distinct Regulatory T Cell Subsets in Colorectal Cancer

**DOI:** 10.1016/j.immuni.2025.11.014

**Published:** 2025-12-15

**Authors:** Xiao Huang, Dan Feng, Sneha Mitra, Emma S. Andretta, Nima B. Hooshdaran, Aazam P. Ghelani, Eric Y. Wang, Joe N. Frost, Victoria R. Lawless, Aparna Vancheswaran, Qingwen Jiang, Cheryl Mai, Karuna Ganesh, Christina S. Leslie, Alexander Y. Rudensky

**Affiliations:** 1 Immunology Program, Sloan Kettering Institute, Memorial Sloan Kettering Cancer Center, New York, NY, USA; 2 Tisch Cancer Institute, Icahn School of Medicine at Mount Sinai, New York, NY, USA; 3 Computational and Systems Biology Program, Sloan Kettering Institute, Memorial Sloan Kettering Cancer Center, New York, NY, USA; 4 Louis V. Gerstner Jr Graduate School of Biomedical Sciences, Memorial Sloan Kettering Cancer Center, New York, NY, USA; 5 Immunology and Microbial Pathogenesis Program, Weill Cornell Graduate School of Medical Sciences, New York, NY, USA; 6 Tri-Institutional MD-PhD Program, Weill Cornell Medicine, The Rockefeller University and Memorial Sloan Kettering Cancer Center, New York, NY, USA; 7 Immunology and Microbial Pathogenesis Program, Weill Cornell Medicine Graduate School of Medical Sciences, New York, NY, USA; 8 Molecular Pharmacology Program, Sloan Kettering Institute, Memorial Sloan Kettering Cancer Center, New York, NY, USA; 9 These authors contributed equally to the manuscript.; 10 Lead contact

## Abstract

Enrichment of regulatory T (Treg) cells in solid organ cancers is generally associated with poor prognosis; however, colorectal cancer (CRC) stands out as a notable exception. Here, we examined the heterogeneity of tumoral Treg cells in CRC and identified two distinct tumoral Treg subsets with differential *Il10* expression. Selective depletion of interleukin-10-expressing (IL-10^+^) Treg cells promoted tumor growth by lifting the restraint on IL-17 production from effector CD4^+^ T cells, thereby directly stimulating tumor cell proliferation; depletion of IL-10^−^ Treg cells led to pronounced tumor regression. In human CRC, IL-10^+^ and IL-10^−^ Treg abundance correlated with favorable and unfavorable prognosis, respectively. Accordingly, IL-10^+^ and IL-10^−^ Treg cells exhibited opposite enrichment patterns in adjacent normal colon tissues and tumors. Transcriptionally similar Treg subsets were observed across different human barrier tissue tumors. This functional dichotomy between Treg subsets may enable selective targeting of the pro-tumoral subset while preserving its anti-tumoral counterpart in CRC and other barrier tissue cancers.

## eTOC blurb

Huang et al study heterogeneity of regulatory T (Treg) cells in a genetic model of colorectal cancer (CRC), and find that interleukin (IL)-10^−^ Treg cells promote tumor progression, whereas IL-10^+^ Treg cells restrain tumor growth by suppressing IL-17 production from CD4^+^ T cells. This functional dichotomy between Treg subsets is seen in human CRC, and may be relevant in other barrier tissue cancers.

## Graphical Abstract

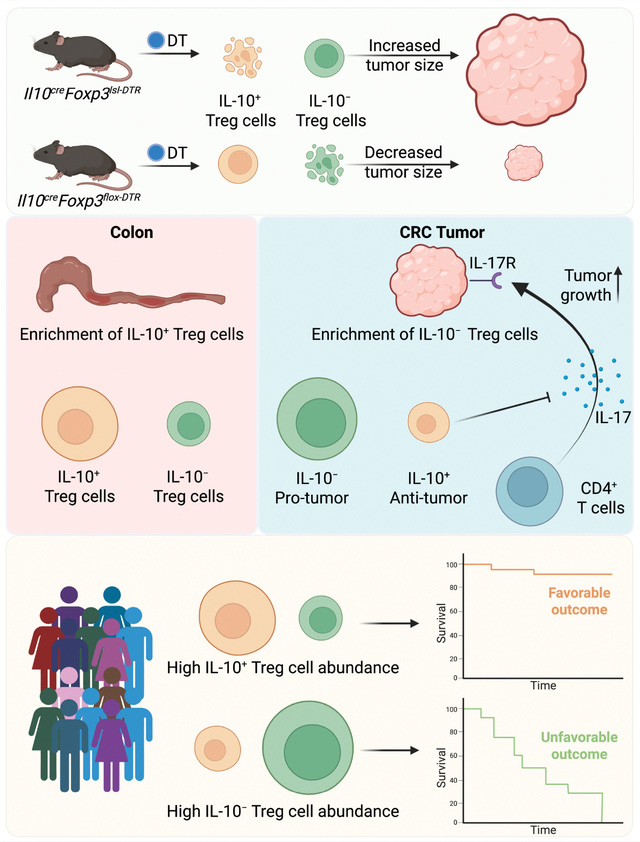

## INTRODUCTION

Regulatory T (Treg) cells, expressing the transcription factor Foxp3, are essential for organismal physiology and fitness. Parallel to the effector arm of adaptive immunity, they patrol the organism through recirculation and residence in secondary lymphoid organs and seed non-lymphoid tissues including gastrointestinal and genital tracts, skin, and airways. In these tissues, exposed to a variety of biotic and abiotic stressors, Treg cells establish and maintain immunological tolerance and limit inflammation through diverse mechanisms.^1,2^ These cells also promote tissue repair during injuries by producing maintenance and differentiation factors and partaking in tissue stem cell niches.^3^

Both the immunosuppressive and tissue-maintenance functions of Treg cells feature prominently in solid organ cancers, where they assume a highly activated state. In the tumor microenvironment (TME), Treg cells regulate other immune and non-immune accessory cells or act directly on tumors to favor their progression. The pronounced enrichment of tumoral Treg cells in solid organ cancers, generally linked to poor prognosis,^4–10^ has inspired recent efforts to target these cells as a means of cancer immunotherapy. Treg cell depletion in experimental cancers, including PD-1 and CTLA-4 blockade-resistant genetically induced tumors and their orthotopically transplanted derivatives, resulted in pronounced inhibition of tumor growth.^11–13^ Unlike most established tumors where Treg cells are tumor-supportive, colorectal cancer (CRC), the second leading cause of cancer-related mortality,^14^ appears to be an exception; there is a positive correlation between Treg and CD8^+^ T cell densities in human CRC tumors and enhanced tumor control.^15^ CRC is generally classified into two primary types: 80–85% of CRC cases are microsatellite stable (MSS) with proficient mismatch repair (MMRp), while the remaining 15% are microsatellite instability-high (MSI-H) with deficient mismatch repair (MMRd). Combined PD-1 and CTLA-4 blockade is effective in MSI-H but largely ineffective in MSS CRC.^16,17^ A notable feature distinguishing the MSS from MSI-H CRC TME is the Treg cell prevalence in the former.^15^ Mouse studies show high heterogeneity of colonic Treg cells that stems from their distinct ontogeny^18–20^, differentiation states, and specific effector features.^21^ The latter may contribute to different aspects of colon tissue biology, exemplified by interleukin-10-expressing (IL-10^+^) Treg cells preventing colon shortening.^21^ These findings raise a possibility that while in MSS CRC some tumoral Treg cells have an expected tumor-promoting function, a distinct subset of tumoral Treg cells may exert a seemingly incongruous anti-tumoral function by restraining activity of tumor-supportive accessory cells.

Here, we tested this supposition by investigating transcriptional and chromatin heterogeneity of tumoral vs colonic Treg cells at single-cell resolution in an orthotopic mouse model of MSS CRC as well as in MSS CRC samples from human patients. Analysis of the dynamics of tumoral Treg population during progression of tumors, established upon transplantation of *Apc*^−/−^*Trp53*^−/−^ *Kras*^*G12D*^ cancer organoids,^22^ showed an enrichment in IL-10-non-expressing (IL-10^−^) Treg cells over IL-10^+^ Treg cells, whereas the opposite pattern was observed in normal colon. Accordingly, targeted depletion of IL-10^+^ Treg cells increased IL-17 production by effector CD4^+^ T cells, thereby promoting CRC growth, while selective loss of IL-10^−^ counterparts resulted in tumor shrinkage. Furthermore, the dichotomy was also conserved in human MSS CRC, where higher IL-10^+^ and IL-10^−^ Treg prevalence correlated with favorable and unfavorable survival, respectively. Meta-analysis of single-cell multiome datasets further identified Treg cell subsets with similar features in human lung and skin cancers. Together, our studies suggest that in MSS CRC and likely other barrier tissue malignancies, tumoral IL-10^+^ Treg cells may exert an indirect antitumoral function by suppressing, in an IL-10-dependent manner, the effector T cell production of IL-17 acting as a tumor growth factor.

## RESULTS

### The AKP tumor is a faithful experimental model of human MSS CRC

**Figure 1.**
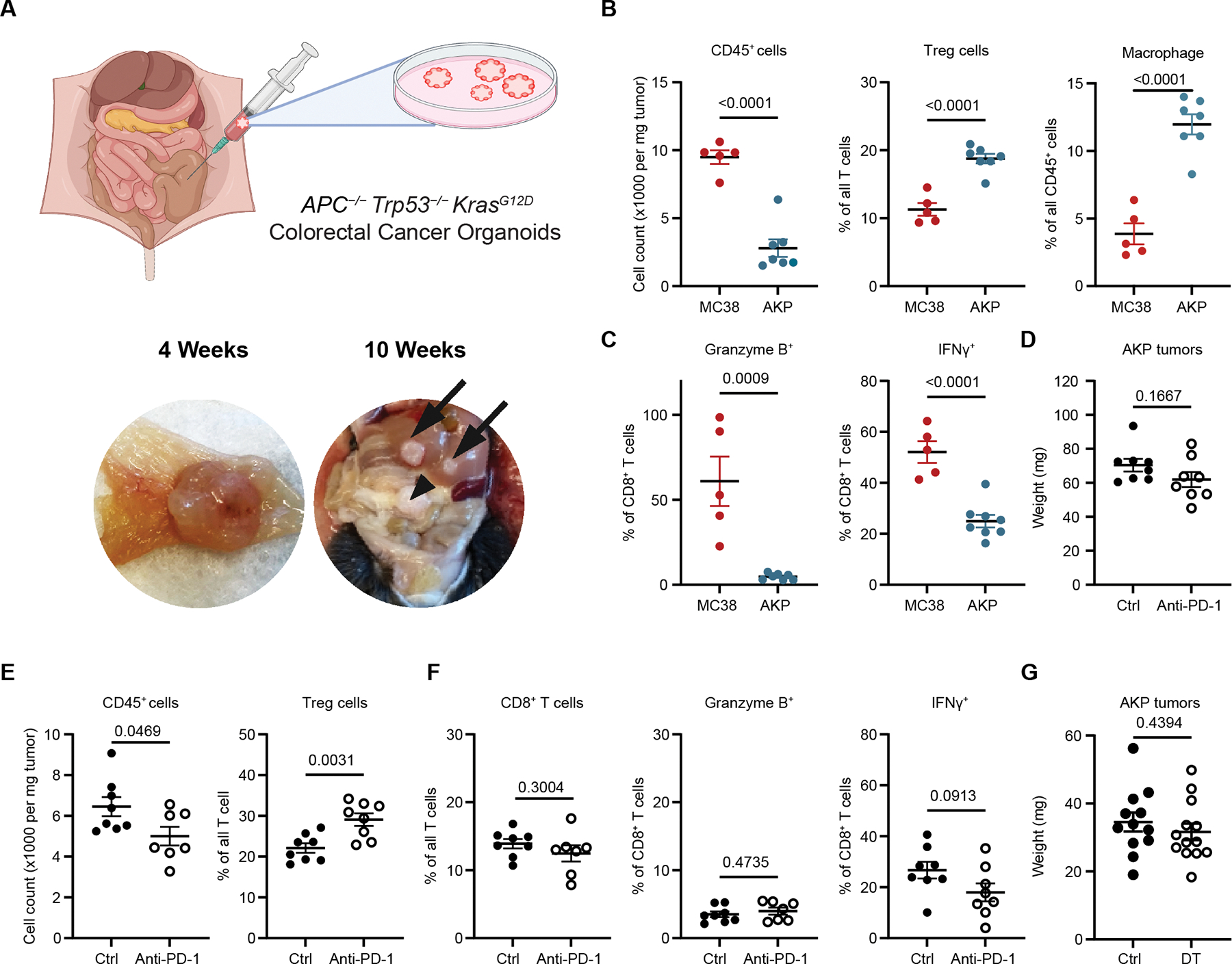
Orthotopic AKP tumor organoid model of human MSS CRC. (**A**) The schematic of orthotopic AKP tumor organoid transplantation model (top); representative images of primary and metastasized tumors (arrows) at indicated time points (bottom). (**B and C**), The frequencies of immune populations (**B**) or cytokine-producing CD8^+^ T cells (**C**) as measured by flow cytometric analysis of the indicated tumors 2 weeks after transplantation; (**D–F**) Analysis of tumor sizes (**D**), frequency of immune populations (**E**), or frequency of and cytokine production by CD8^+^ T cells (**F**) at the end of isotype control (Ctrl) or PD-1 antibody (Anti-PD-1) treatments. (**G**) Weights of AKP tumors two weeks after implantation with DT or bDT (Ctrl) treatment. Data are pooled from 2–3 independent experiments and are shown as mean ± s.e.m. Statistical analysis was performed using unpaired *t*-tests.

To investigate the heterogeneity and function of Treg cells in MSS CRC, we employed an orthotopic mouse CRC model that recapitulates human disease properties, including hallmark CRC driver mutations, anatomic features, TME immune cell composition, abundant Treg cell and resistance to PD-1 blockade. Orthotopic transplantation of mouse organoids derived from intestinal stem cells with the induced *Apc* and *Trp53* gene loss and oncogenic *Kras*^*G12D*^ expression (AKP)^22^ gave rise to tumors, morphologically resembling human CRC; AKP tumors protruded into the lumen and were macroscopically detectable at 2–4 weeks with spontaneous metastases observed in the draining mesenteric lymph nodes (mLNs) and the liver at 10 weeks after implantation of organoid cells into the cecal wall (Figure 1A). Notably, the AKP TME showed overall significantly reduced immune cell presence with an enrichment in Treg cells and macrophages, and diminished effector CD8^+^ T cell activities in comparison to hypermutated MC38 colon adenocarcinoma, a commonly used mouse model of MSI-H CRC (Figures 1B and 1C).^23^ The observed features distinguishing these orthotopic mouse models closely aligned with those of corresponding human CRC types (Figure S1),^24^ highlighting the pronounced immunosuppressive character of the AKP tumor model.^6^ Contrary to antibody-mediated PD-1 blockage sensitivity of MC38,^25^ AKP tumors were resistant to this therapeutic modality, which resulted in a mild increase in Treg cells with no significant changes in tumor volume or CD8^+^ T cell activation status (Figures 1D–1F). This mirrored the therapeutic response to PD-1 blockade and the lack thereof in the corresponding human CRC types. Together, these initial experiments established the AKP tumor as a faithful model of human MSS CRC.

### AKP tumor associated Treg and effector T cell heterogeneity

Considering that tumors regress upon Treg depletion in most experimental mouse models and that human MSS CRC deviates from a typical association of tumoral Treg “richness” with poor prognosis, we investigated the efficacy of therapeutic Treg targeting in AKP tumor-bearing *Foxp3*^*DTR*^ mice. In this mouse model, diphtheria toxin receptor (DTR) is expressed under the control of the endogenous *Foxp3* locus, which enables punctual removal of Treg cells upon diphtheria toxin (DT) administration. Consistent with the reported lack of a clear link between Treg abundance and clinical outcome in CRC patients, we found that the DT-induced pan-Treg depletion after AKP tumor organoid implantation did not significantly alter tumor growth (Figure 1G). Although these results seemingly suggested Treg functionality is dispensable for tumor outcome, it remained possible that distinct tumoral Treg subsets may exert opposing effects on tumor growth resulting in a zero-sum effect.

**Figure 2.**
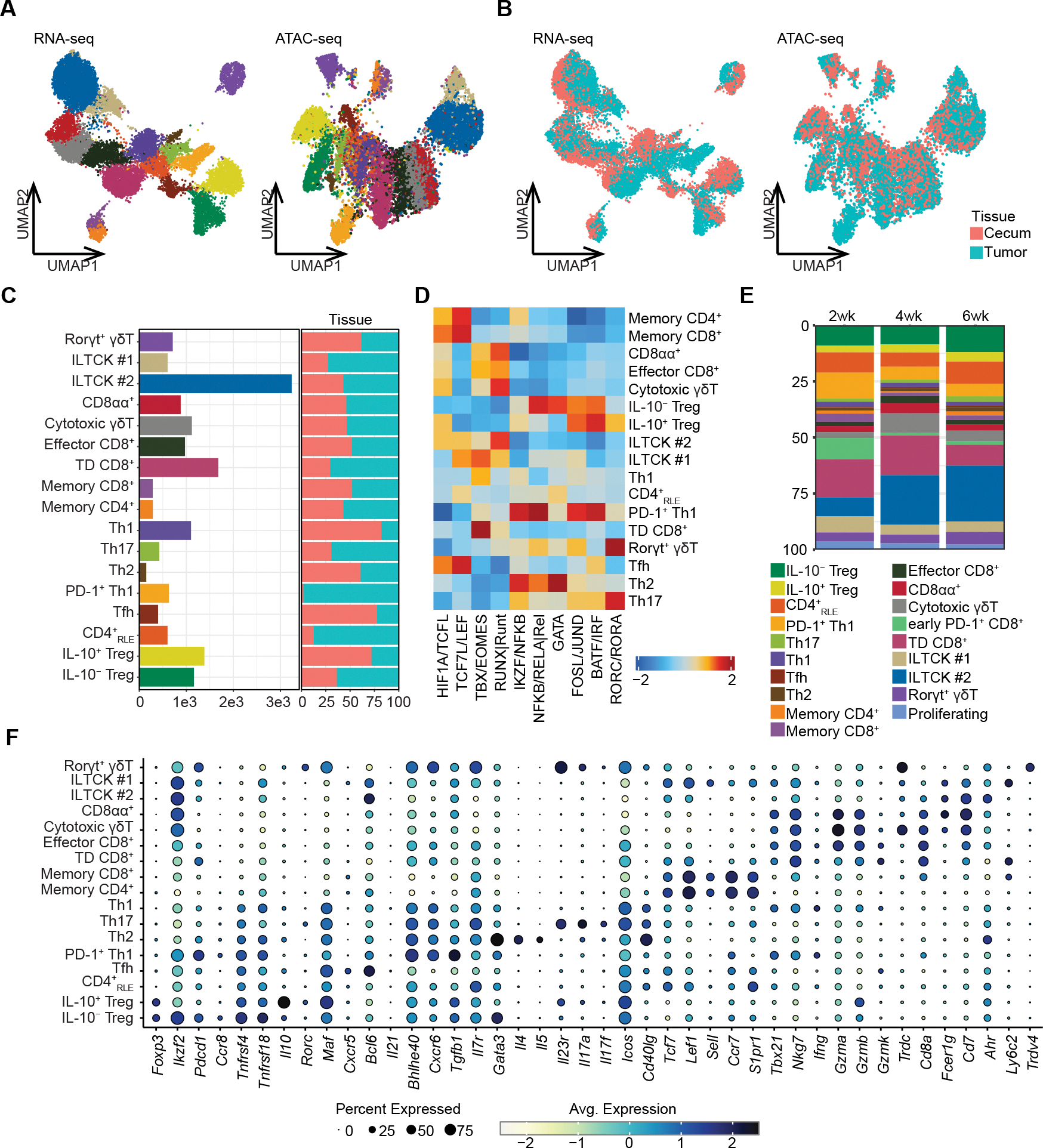
Paired single-cell RNA-seq and ATAC-seq analysis of CRC tumor associated T cells. (**A–C**), UMAP plots of paired scRNA-seq and scATAC-seq analysis of AKP-tumor-associated T cells depicting cell type annotations (**A**) and tissue annotations (**B**) (n=15,621 cells). Bar graphs depicting the number of cells for each cell type and the proportions for each tissue (**C**). (**A**) and (**C**) share the same color scheme for each population. (**D**) chromVAR motif enrichment analysis of multiome scATAC-seq data across different T cell populations. (**E**) The tumoral T cell subset dynamics 2, 4, and 6 weeks after tumor implantation determined by scRNA-seq (n=21,269 cells total: 10,013 from normal adjacent cecal tissues and 11,256 from AKP tumors). (**F**) The expression of selected genes of interest in AKP tumoral T cell subsets 4 weeks after tumor inoculation. TD CD8^+^: terminally differentiated CD8^+^ T cells. CD4^+^_RLE_: recent LN emigrant CD4^+^ T cells.

To address this possibility, we first performed paired single-cell RNA/ATAC-seq analyses of purified T cell populations from primary AKP tumors and adjacent tumor-free cecal tissues 4 weeks post-transplantation. 17 distinct clusters (15,621 cells) were identified based on the latent space representation of scRNA-seq data and visualized using uniform manifold approximation and projection (UMAP) (Figures 2A–2C). Analysis of the effector T cells showed that CD8^+^ T cells encompassed three clusters of CD8αβ^+^ T cells, containing terminally differentiated (TD CD8^+^, *Pdcd1*^hi^ *Gzmb*^lo^), effector (*Gzma*^hi^ *Gzmb*^hi^), and memory (*Il7r*^hi^ *Ccr7*^hi^) CD8αβ^+^ T cells, and one cluster of CD8αα^+^ T cells (Figure 2F). Effector CD4^+^ T cells encompassed two Th1 (*Tbx21*^+^ *Ifng*^+^) clusters, alongside Th2 (*Gata3*^+^ *Il4*^+^ *Il5*^+^), Th17 (*Rorc*^+^ *Il17a*^+^ *Il17f*^+^), Tfh (*Cxcr5*^+^ *Bcl6*^+^), memory (*Ccr7*^hi^ *Sell*^hi^ *Il7r*^hi^), and recent draining LN emigrant (CD4^+^RLE, *S1pr1*^hi^) clusters (one each). Accordingly, analysis of ATAC-seq peaks in each cluster showed enrichment of corresponding lineage-defining transcription factor motifs (TBX for Th1 and effector and TD CD8^+^ T cells; SOX/TCF for memory cells; GATA for Th2s; ROR for Th17) (Figure 2D). Beyond classical αβT cells, we also identified two clusters of *Rorc*^+^ or cytotoxic *Gzma*^hi^ *Gzmb*^hi^ γδ T cell, as well as two recently described innate-like *Fcer1g*^+^ T cells (ILTCKs) clusters.^26^ Most of the identified clusters contain both tumoral and colonic T cells, however, one cluster of highly activated PD-1^+^ Th1 cells appears to reside only in tumors (Figures 2C and 2F). Importantly, the effector T cell clusters identified in AKP tumors closely paralleled those observed in human MSS CRCs,^24^ lending further support for the exactitude of this model of human disease.

Treg cells formed two clusters, annotated as IL-10^+^ and IL-10^−^ Treg cells based on *Il10* expression. Both were populated by colonic and tumoral cells, but most IL-10^+^ Treg cells were in the cecum. In contrast, IL-10^−^ Treg cells were enriched in the tumor, raising a possibility of potentially distinct functions of IL-10^+^ and IL-10^−^ Treg cells in MSS CRC (Figure 2C). To gain insights into their dynamics during AKP tumor progression, we performed scRNA-seq of T cell populations from tumors and adjacent cecal tissues at 2, 4, and 6 weeks after AKP organoid implantation. All 17 clusters identified in the multiome analysis were captured by the time course scRNA-seq analysis, which also identified two additional early *Pdcd1*^hi^ CD8^+^ and proliferative T cell clusters (Figure 2E). Extending the observation of the tumoral Treg subset composition bias revealed by single-cell multiome analysis, the scRNA-seq time course, and corroborating flow cytometric analyses, showed an increasing enrichment of IL-10^−^ relative to IL-10^+^ Treg cells in tumors but not adjacent normal cecal tissues, while the frequency of IL-10^+^ Treg cells remain unchanged (Figures 2E, S2A, and S2B). The increase in IL-10^−^ Treg abundance was accompanied by a pronounced decline in tumor-specific PD-1^+^ Th1 cells and early *Pdcd1*^hi^ CD8^+^ T cells. These two populations of effector cells were most abundant in early tumors at 2 weeks post-implantation but not present in normal colonic tissue (Figures 2E and S2A).

The dynamic of IL-10^+^ and IL-10^−^ Treg cells raised the question of their plasticity. To address this, we “time-stamped” preexisting Treg pool through YFP induction in *Foxp3*^*CreER*^*Rosa26*^*lsl-YFP*^ mice pulsed with tamoxifen prior to tumor implantation. 14 days after AKP tumor organoid implantation, almost all tumor infiltrating Treg cells remain YFP^+^ indicating that essentially the entire tumoral Treg population arose from the migration and expansion of preexistent Treg cells while their *de novo* generation was minimal (Figure S2C). To determine whether IL-10^+^ and IL-10^−^ Treg cells can interconvert, we employed analogous approach of fate mapping of IL-10 expressing Treg cells in tamoxifen-pulsed *Il10*^*CreER*^*Rosa26*^*lsl-YFP*^*Foxp3*^*Thy1.1*^ mice. Previous study showed that in the normal colon IL-10^+^ Treg cells represent a stable terminally differentiated cell state.^21^ Consistently, we found that the majority of YFP tagged pre-existing IL-10^+^ Treg cells remained IL-10^+^ in both AKP tumors and the adjacent normal cecal tissue as well as draining lymph nodes (LNs) (Figure S2D). Notably, two weeks after tumor implantation the majority of IL-10^+^ Treg cells were newly generated as they lacked YFP expression, suggesting rapid turnover of IL-10^+^ Treg cells and their replenishment by their activated IL-10^−^ counterparts (Figure S2E). Together, these observations were consistent with the proposed distinct functions of two Treg cell subsets, prompting their experimental assessment.

### Divergent gene expression programs of tumoral IL-10^+^ and IL-10^−^ Treg cell subsets

**Figure 3.**
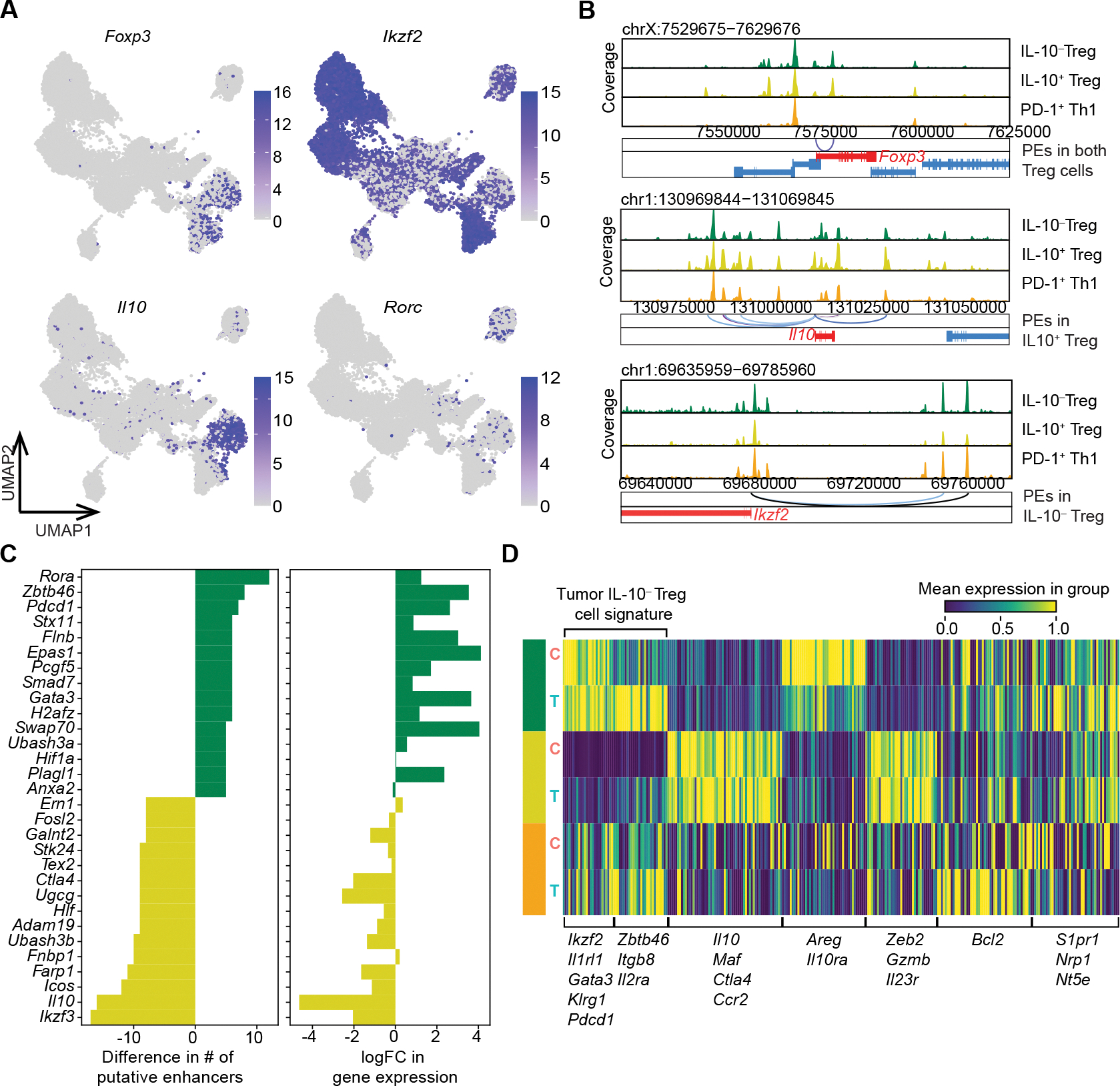
Heterogeneity of CRC tumoral Treg cells. (**A**) UMAP plots depicting the expression of marker genes distinguishing IL-10^+^ and IL-10^−^ Treg cells. (**B**) Gene browser chromatin accessibility tracks for *Foxp3*, *Il10*, and *Ikzf2* gene loci for IL-10^+^ and IL-10^−^ Treg cells depicting putative enhancers (PEs) linked to transcription start site for each gene. (**C**) Numbers of PEs (left) and log fold change in gene expression (right) for select genes for IL-10^+^ and IL-10^−^ Treg cells. (**D**) Heatmap depicting expressions of gene modules identified across all Treg subsets split by tumor (T) and adjacent tumor-free cecum (C).

The dynamic of IL-10^+^ and IL-10^−^ Treg cell subsets in CRC implied their distinct gene regulatory and transcriptional features. In this context, the former subset predominantly expressed *Rorc* gene, which encodes the transcription factor RORγt, whereas the latter expressed *Ikzf2* transcripts encoding Helios (Figure 2F). Thus, despite the presence of relatively minor fractions of RORγt^+^ IL-10^−^ and Helios^+^ IL-10^+^ Treg cells, RORγt^+^ and Helios^+^ Treg cells can serve as surrogates for the corresponding IL-10^+^ and IL-10^−^ Treg cell subsets in the AKP tumors and adjacent normal colonic tissues (Figure 3A). To further dissect their divergent transcriptional regulation, we employed the SCARlink algorithm to identify putative enhancers of signature genes for both populations by analyzing paired ATAC- and RNA-seq datasets.^27^ Pseudo-bulk ATAC-seq tracks demonstrated differential accessibility between IL-10^+^ vs IL-10^−^ Treg cells at the putative enhancer sites in the *Il10* and *Ikzf2* loci, but not the *Foxp3* locus, reflecting their distinct transcriptional regulatory circuitry (Figure 3B). Consistently, the difference in the number of gene-linked enhancers for some of the top differentially expressed genes closely correlated with the expression of the corresponding transcripts in these two subsets (Figure 3C).

To identify transcriptional modules expressed by IL-10^+^ vs IL-10^−^ Treg cells, we employed Hotspot,^28^ and identified gene modules unique to each annotated Treg subset. Some of these modules also displayed tumor- or cecum-specific patterns indicating potential environmental imprinting of these programs (Figure 3D). Among the gene modules specific to IL-10^−^ but not IL-10^+^ Treg cells, two showed equal or higher expression in cells residing in AKP tumors when compared to their cecal counterparts. Henceforth referred to as the tumor IL-10^−^ Treg gene signature, these modules comprised either *Ikzf2*, *Il1rl1*, *Gata3*, and *Klrg1* transcripts, or *Zbtb46*, *Itgb8*, and *Il2ra* transcripts. In contrast, another IL-10^−^ Treg specific gene module with characteristic *Areg* and *Il10ra* expression, was preferentially detected in cells residing in the adjacent normal colonic tissues. This observed expression pattern likely reflected the heightened states of activation of tumoral IL-10^−^ Treg cells and the tissue-supporting function of their colonic counterparts. While two separable gene modules were also identified as IL-10^+^ Treg-specific, their expression did not vary between the tumors and adjacent normal colonic tissues, indicating the lack of noticeable modulation of IL-10^+^ Treg transcriptional features by the CRC TME. These two IL-10^+^ Treg-specific gene modules were characterized by enrichment in *Il10, Maf, Ctla4,* and *Ccr2* transcripts, and *Zeb2, Gzmb,* and *Il23r* transcripts, respectively (Figure 3D). Analysis of the ATAC-seq data using chromVar showed that while IL-10^+^ and IL-10^−^ Treg cells have shared enrichment of certain TF binding motifs at their unique accessible chromatin sites, the NR4A family motif was selectively enriched in tumoral IL-10^−^ Treg cells (Figure S3A). This result suggested stronger TCR signaling experienced by the tumor-residing IL-10^−^ Treg cells in agreement with their heightened activation state implied by RNA-seq analysis (Figure 3D). In support of this notion, gene score analysis of tumoral IL-10^+^ Treg, IL-10^−^ Treg, and PD-1^+^ Th1 cells showed the highest TCR signaling-related gene expression in tumoral IL-10^−^ Treg cells among all three subsets. (Figure S3B). Collectively, these results suggested functional divergence between tumoral IL-10^+^ and IL-10^−^ Treg cells.

### IL-10^+^ and IL-10^−^ Treg cells have opposing functions in MSS CRC

Previous studies have implicated IL-33 receptor ST2 (encoded by *Il1rl1*) in repressing IL-17 production by CRC Treg cells^29–31^. Since *Il1rl1* was among IL-10^−^ Treg signature genes, we further examined the expression of transcripts encoding ST2 and IL-17 family members by both subsets. While *Il1rl1* was exclusively co-expressed with *Pdcd1* by IL-10^−^ Treg cells, we detected minimal expression of *Il17a* and *Il17f* by either subset of Treg cells (Figure S3C) with no changes in the expression of *Pdcd1* and other major inhibitory receptors over the course of 6 weeks (Figure S3D). These observations, together with the reported dispensability of ST2 in healthy non-lymphoid tissues and colonic tumors,^32,33^ indicate that Treg production of IL-17 has minor contribution to their function in the AKP model of CRC.

**Figure 4.**
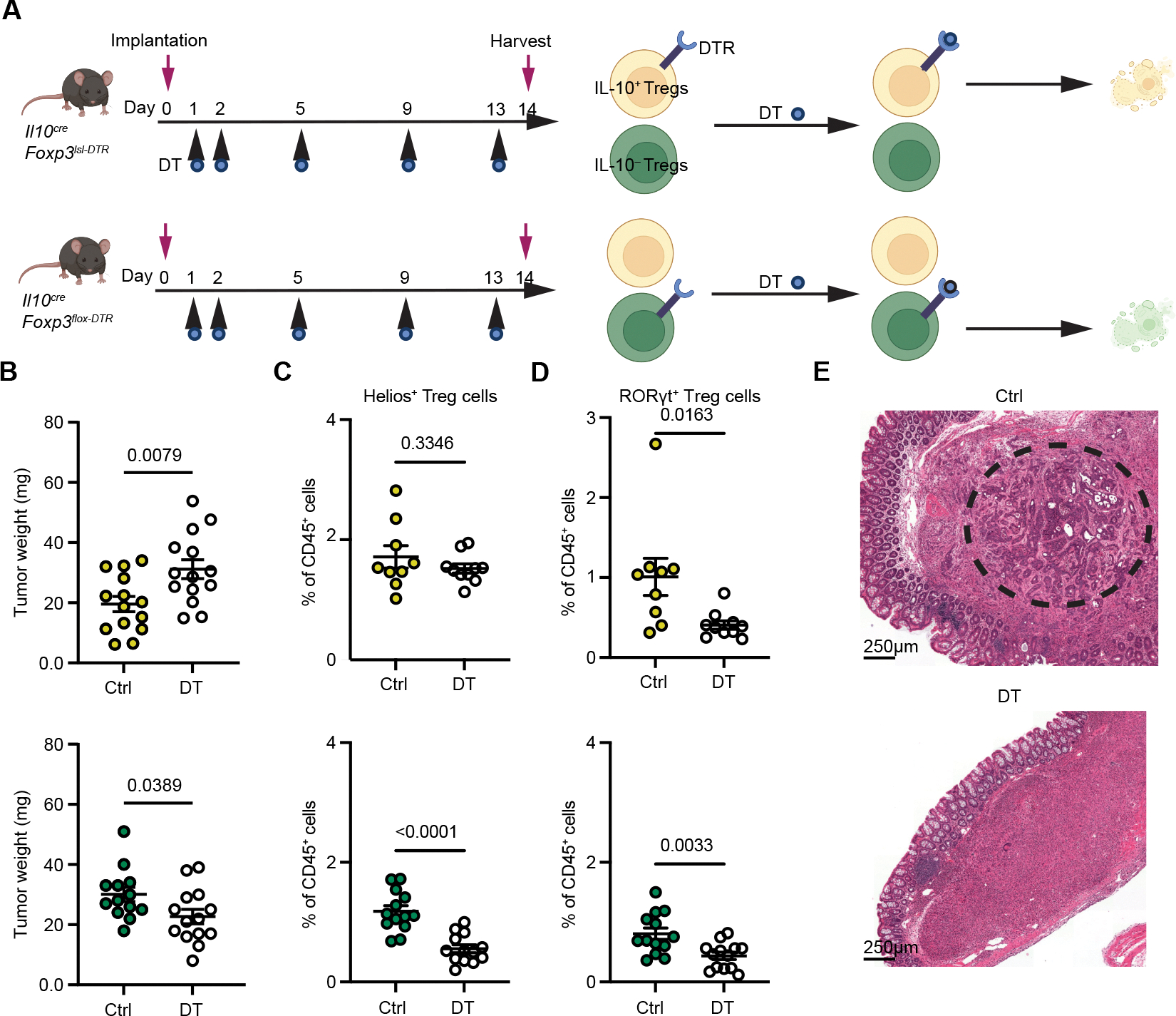
Targeted ablations of IL-10^+^ or IL-10^−^ Treg cells result in opposing effects on CRC tumor growth. (**A**) The experimental design schematic of diphtheria toxin (DT) induced selective ablation of IL-10^+^ and IL-10^−^ Treg cells using genetic mouse models. (**B**) Tumor sizes in DT- and control (Ctrl) bDT-treated *Il10*^*Cre*^ *Foxp3*^*lsl-DTR*^ (top) and *Il10*^*Cre*^ *Foxp3*^*flox-DTR*^ mice (bottom) two weeks after AKP organoid implantation. (**C** and **D**) Flow cytometric analyses of the frequencies of Helios^+^ Treg (**C**) and RORγt^+^ Treg (**D**) populations in DT and Ctrl bDT-treated *Il10*^*Cre*^ *Foxp3*^*lsl-DTR*^ (top) and *Il10*^*Cre*^ *Foxp3*^*flox-DTR*^ mice (bottom). (**E**) H&E staining of AKP tumors from indicated treatment groups. Images are representative of 3 independent experiments (dashed circle in the left panel indicates the area of the tumor cells (dark purple cells). For (**B–D**) pooled data from two independent experiments are shown as mean ± s.e.m. Statistical analysis was performed using unpaired *t*-tests.

To directly investigate the functionality of IL-10^+^ and IL-10^−^ Treg subsets in the AKP tumor model, we employed their punctual selective depletion using two complementary genetic strategies. IL-10^+^ Treg cells were ablated upon DT administration into AKP tumor-bearing *Il10*^*tdTomato-Cre*^*Foxp3*^*lsl-DTR*^ (*Il10*^*Cre*^*Foxp3*^*lsl-DTR*^) mice, in which *Il10* locus-encoded Cre recombinase excised a *loxP* site-flanked STOP cassette preceding the DTR coding sequence inserted into the *Foxp3* locus. Consequently, DTR is expressed in IL-10^+^ Treg cells in these mice. As a complementary approach, we induced ablation of IL-10^−^ Treg cells in AKP tumor-bearing *Il10*^*tdTomato-Cre*^*Foxp3*^*flox-DTR*^ (*Il10*^*Cre*^*Foxp3*^*flox-DTR*^) mice, in which *Il10* locus-encoded Cre excised the *loxP* site-flanked DTR coding sequence from the *Foxp3* locus, resulting in DTR expression in IL-10^−^ Treg cells. The experimental and control groups of mice were treated with DT or heat-inactivated DT (boiled DT; bDT), respectively, on day 1, 2, 5, 9, and 13 after intracecal implantation of AKP tumor organoids (Figure 4A). This resulted in efficient removal of DTR-expressing Treg populations in *Il10*^*Cre*^*Foxp3*^*lsl-DTR*^ and *Il10*^*Cre*^*Foxp3*^*flox-DTR*^ mice (Figure S4A), as indicated by the pronounced preferential depletion of tumoral RORγt^+^ and Helios^+^ Treg cells, respectively (Figures 4C, 4D, S4B, and S4C). The highly specific depletion of RORγt^+^ Treg cells in *Il10*^*Cre*^*Foxp3*^*lsl-DTR*^ was consistent with our observation that colonic IL-10^+^ Treg cells are highly enriched for RORγt expression and represent a stable, terminally differentiated cell state (Figures S2D, 3A). A concomitant decrease in RORγt^+^ Treg cells observed in DTtreated *Il10*^*Cre*^*Foxp3*^*flox-DTR*^ mice was likely due to the lack of *Il10* expression by some RORγt^+^ Treg cells and the robust replenishment of IL-10^+^ Treg cells by their IL-10^−^ counterparts (Figures 3A and S2E).^21^ While these Treg subset perturbations did not significantly alter overall immune cell infiltration of AKP tumors (Figure S4D), IL-10^−^ Treg depletion led to a reduction in the tumor size, which upon closer histological and flow cytometrical examination showed negligible remaining tumor cell presence accompanied by pronounced increase in mononuclear cells and tumoral CD8^+^ T cell responses (Figures 4B, 4E, and S4E). This observation suggests efficient mobilization of anti-tumor effectors upon removal of IL-10^−^ Treg cells. The heightened expression of *Pdcd1* distinguishing pro-tumoral IL-10^−^ Treg cells likely contributes to their expansion upon PD-1 blockade and the PD-1 targeted therapy resistance observed for AKP tumors and previously reported for human MSS CRC (Figure S3D). In contrast, IL-10^+^ Treg cell depletion increased tumor size by 50% without detectable changes in effector CD8^+^ or CD4^+^ T cell frequencies, suggesting the enhanced tumor burden was not a consequence of diminished overall anti-tumoral T cell response, but its altered “flavor” worsening disease course (Figures 4B and S4E).

**Figure 5.**
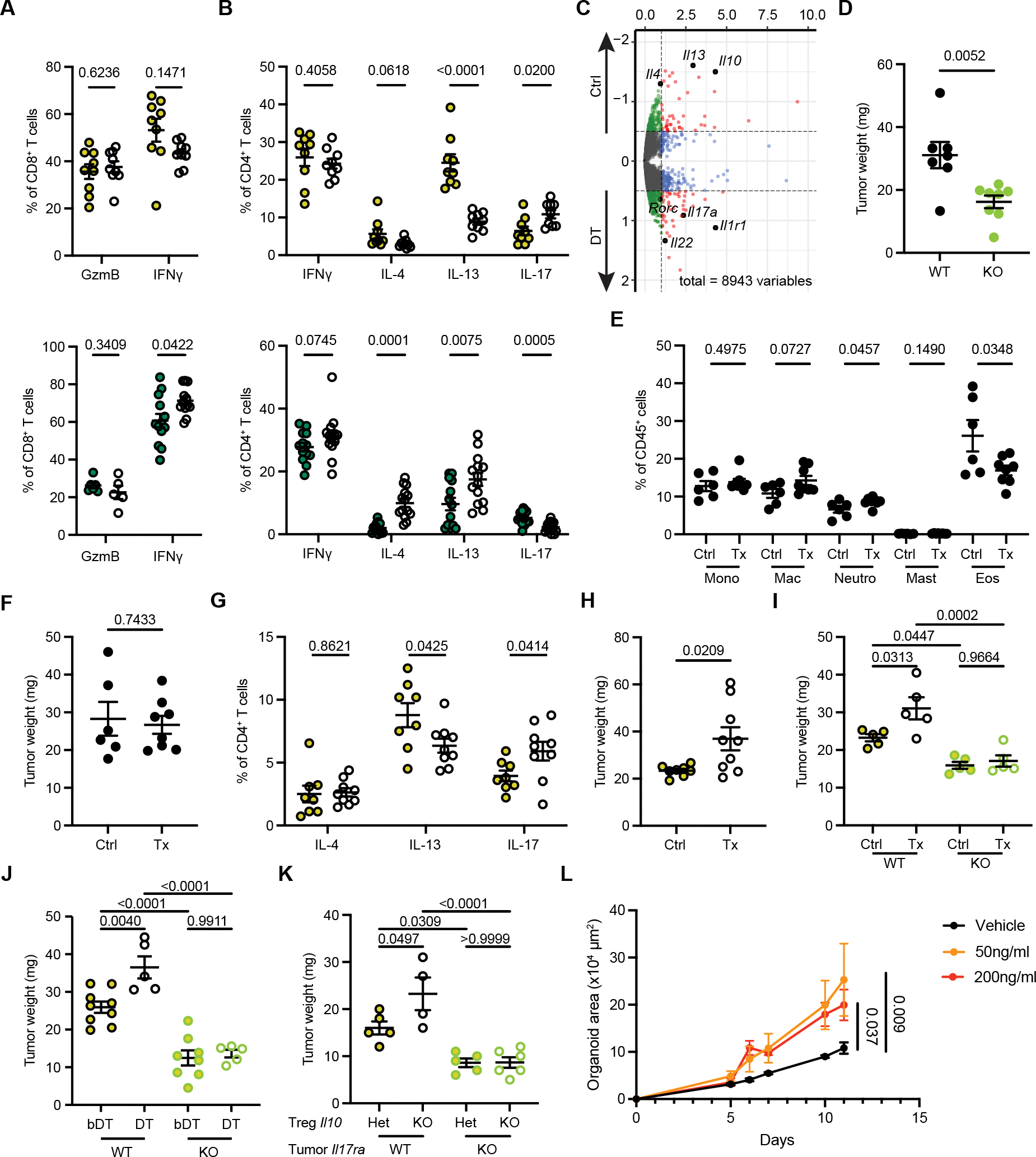
IL-10^+^ and IL-10^−^ Treg cells exert anti-and pro-tumoral functions through regulation of CD4^+^ T cell responses. (**A and B**) Flow cytometric analyses of the indicated cytokines production by PMA/ionomycin stimulated CD8^+^ (**A**) and CD4^+^ T cells (**B**) isolated from AKP tumors from *Il10*^*Cre*^ *Foxp3*^*lsl-DTR*^ (top) and *Il10*^*Cre*^ *Foxp3*^*flox-DTR*^ mice (bottom) two weeks after implantation. (**C**) Pseudo-bulk RNA-seq analysis of differential gene expression in tumoral CD4^+^ T cells from DT vs bDT (Ctrl) treated *Il10*^*tdTomato-Cre*^ *Foxp3*^*lsl-DTR*^ mice two weeks after implantation. Key genes of interest are labeled. (**D**) Weights of *Il17ra*-sufficient (WT) and -deficient (KO) AKP tumors two weeks after implantation into C57BL/6 mice. (**E–H**) Flow cytometric analyses of the indicated cell types (**E**) or indicated cytokines production by PMA/ionomycin stimulated CD4^+^ T cells (**G**), and tumor weights (**F and H**) two weeks after the indicated treatments; for (**E**) and (**F**), Tx: IL-4 and IL-13 neutralizing antibodies; Ctrl: isotype control IgG; for (**G**) and (**H**), Tx: IL-10Rα blocking antibody; Ctrl: isotype control IgG. (**I–K**) Weights of *Il17r*-sufficient (WT) and -deficient (KO) AKP tumors two weeks after their implantation into C57BL/6 mice (**I**), *Il10*^*Cre*^ *Foxp3*^*lsl-DTR*^ mice (**J**), or lethally irradiated T cell-deficient mice reconstituted with *Foxp3*^*CreER*^*Il10*^*flox/wt*^ (Het) or *Foxp3*^*CreER*^*Il10*^*flox/flox*^ BM cells (KO) (**K**) and receiving indicated treatments. For (**I**), Tx: IL-10Rα blocking antibody; Ctrl: isotype control IgG. (**L**) Organoid growth as measured by total area covered by organoid cells over all Z-stack. For (**A and B**), and (**D–K**), data were pooled from two independent experiments and are shown as mean ± s.e.m. For (**L**), data were pooled from three independent cultures and are shown as mean ± s.e.m. For (**A and B**), and (**D–H**), statistical analysis was performed using unpaired *t*-tests. For (**I–K**), statistical analysis was performed using one-way ANOVA. For (**L**), statistical analysis was performed using two-way ANOVA.

To characterize potentially divergent effects of DT mediated depletion of these Treg subsets on the AKP TME, we first examined the myeloid populations in AKP tumors 2 weeks after implantation of tumor organoids in *Il10*^*Cre*^*Foxp3*^*lsl-DTR*^ and *Il10*^*Cre*^*Foxp3*^*flox-DTR*^ mice. Ablation of IL-10^+^ Treg cells resulted in a significant increase in Ly6C^hi^ monocytes, macrophages, and neutrophils, but not mast cells or eosinophils, indicative of a heightened type 3 response (Figure S5A, top panels). Tumor-associated macrophages (TAMs) and neutrophils are known for their tumor-promoting functions in CRC.^34–36^ Consistently, we observed an increased frequency of Spp1^+^ TAMs following depletion of IL-10^+^ Treg cells in our scRNA-seq dataset of tumoral non-T cell immune cells isolated from bDT- (Ctrl) and DT-treated *Il10*^*Cre*^*Foxp3*^*lsl-DTR*^ mice (Figure S5C and S5D). Notably, among all major myeloid populations, only tumor-supporting Spp1^+^ macrophages exhibited significant transcriptional changes upon IL-10^+^ Treg ablation, including upregulation of genes encoding immunomodulatory proteins such as *Cd274*, *Ptgs2*, and *Il1rn*, suggesting potential regulation of Spp1^+^ TAMs by IL-10^+^ Treg cells (Figure S5E). In contrast, IL-10^−^ Treg ablation following tumor inoculation resulted in decreased monocyte, macrophage, and neutrophil abundances in the TME, accompanied by an increase in eosinophils, potentially reflective of an increased type 2 response (Figure S5A, bottom panels). Indeed, IL-10^+^ Treg depletion led to a pronounced increase in *Rorc* and type 3 cytokine (IL-17 and IL-22) expression by CD4^+^ T cells with a concomitant decrease in type 2 cytokines (IL-4 and IL-13), whilst granzyme B or IFNγ expression by CD8^+^ T cells was unchanged (Figures 5A and 5B, top panels; 5C and S5B). In a conspicuous contrast, depletion of IL-10^−^ Treg cells led to an increase in IFNγ producing CD8^+^ T cells and type 2 cytokine producing Th2 cells coupled with a decrease in IL-17 expressing Th17 cells (Figures 5A and 5B, bottom panels). Together, these data demonstrate that in AKP tumors IL-10^+^ Treg cells selectively repressed Th17 responses, whereas IL-10^−^ Treg cells repressed Th2 and CD8^+^ T cell responses, suggesting that both subsets modulate the “tone” of tumoral immune response.

### IL-10^+^ Treg cells restrain CRC tumor by limiting IL-17 production

Previous studies implicated IL-17 signaling in promoting early stages of transformation of intestinal epithelial cells in addition to vascularization and recruitment of pro-tumoral myeloid cells.^37–40^ Therefore, the increases in IL-17 and IL-22 and tumor size noted upon depletion of IL-10^+^ Treg cells raised a question whether IL-17 or IL-22 could affect AKP tumors and whether the effect is direct. In support of a specific and direct activity of IL-17, we observed an increased growth of AKP tumor organoids *in vitro* in the presence of IL-17, but not IL-22 (Figure S6). Analysis of the published bulk RNA-seq datasets revealed high *Il17ra* gene expression in similar mouse CRC organoids, indicating that IL-17 may signal directly to AKP tumor cells to promote their proliferation.^41^ To test this hypothesis, we generated IL-17Ra-deficient AKP organoids using CRISPR-Cas9 mediated gene editing. Two weeks after implantation into the cecum, IL-17Ra-deficient tumors were significantly smaller than controls, implicating direct signaling through IL-17RA in IL-17-dependent growth of AKP tumors *in vivo* (Figure 5D). Because tumor growth or restraint from depleting IL-10^+^ or IL-10^−^ Treg cells, respectively, was also associated with opposing effects on IL-4 and IL-13 production, we tested whether a decrease in these cytokines contributed to the increased tumor burden in the absence of IL-10^+^ Treg cells. While antibody mediated neutralization of IL-4 and IL-13 resulted in a two-fold reduction in tumoral eosinophils with a concomitant mild increase in tumoral neutrophils (Figure 5E), tumor size was unaffected in comparison to IgG isotype controls (Figure 5F). These data suggest that a decrease in type 2 cytokines is unlikely to contribute in a non-redundant manner to increased CRC tumor burden in the absence of IL-10^+^ Treg cells.

Previous studies showed that Th17 cells can be directly controlled through IL-10R signaling in response to IL-10 produced by both Foxp3^+^ and Foxp3^−^ CD4^+^ T cells.^42–44^ In light of these observations, our results suggest that IL-10^+^ Treg depletion accelerates tumor growth through IL-17R signaling in AKP cells, induced by increased IL-17 production once IL-10R-mediated restraint is lifted. To test this, we employed antibody-mediated blockade of IL-10Rα signaling in AKP tumor-bearing mice. IL-10Rα blockade resulted in an increase in IL-17 production by CD4^+^ T cells and in tumor sizes similar to those observed upon IL-10^+^ Treg depletion (Figures 5G and 5H). Furthermore, IL-17Ra-deficient AKP tumors were insensitive to IL-10Rα blockade (Figure 5I). Likewise, IL-17Ra-deficient AKP tumors did not respond to DT-mediated IL-10^+^ Treg cell depletion in *Il10*^*cre*^ *Foxp3*^*lsl-DTR*^ mice, indicating Treg cells as major IL-10 producers in the colon (Figure 5J). To further test that IL-10 produced by Treg cells was capable of suppressing IL-17 production by CD4^+^ T cells and restraining tumor burden, IL-17Ra-sufficient (WT) and -deficient (KO) AKP tumors were transplanted into chimeric mice generated upon reconstitution of lethally irradiated T cell deficient mice with *Foxp3*^*CreER*^*Il10*^*flox/wt*^ (Het) or *Foxp3*^*CreER*^*Il10*^*flox/flox*^ (KO) BM cells. Treg-specific ablation of a conditional *Il10* allele induced upon tamoxifen administration 9 and 6 days prior to tumor implantation resulted in significant increase in the size of IL-17Ra-sufficient, but not IL-17Ra-deficient tumors.

Taken together, these results suggest that while tumoral IL-10^−^ Treg cells expectedly promote CRC progression, IL-10^+^ Treg cells restrain primary CRC tumors in an IL-10 signaling dependent manner; Treg-derived IL-10 limits production of IL-17, which acts directly on CRC tumor cells to promote their growth.

### The duality of Treg populations is conserved in human CRC

**Figure 6.**
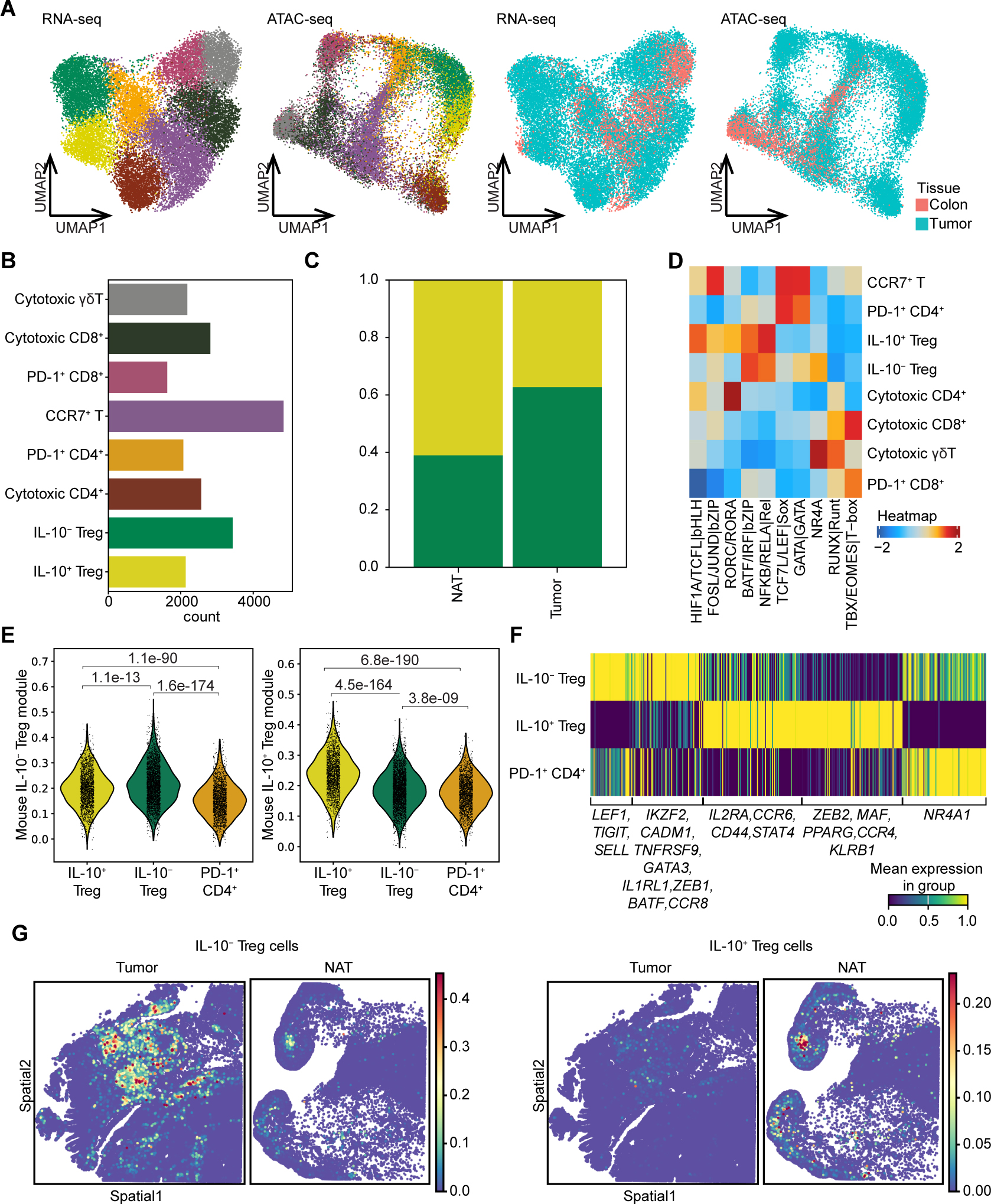
The IL-10^+^ and IL-10^−^ Treg subsets are present in human CRC. (**A**) Paired scRNA-seq and scATAC-seq analysis of T cells isolated from surgical samples of tumors and normal adjacent colon tissues (NATs) of 3 CRC patients. UMAP plots are shown for cell type annotations (left) as indicated in (**B**) and tissue annotations (right) for n=21,676 cells. (**B**) Bar graph depicting the number of cells for each cell type. (**C**) Proportions of IL-10^+^ and IL-10^−^ Treg cells among all Treg cells in indicated tissues. (**D**) chromVAR analysis of differential motif enrichment within scATAC-seq peaks across T cell subsets. (**E**) Violin plots of gene scores calculated on the cells from human multiome analysis using indicated gene modules identified in Figure 3D. (**F**) Heatmap depicting expression of gene modules identified across indicated tumoral T cell subsets from CRC patients. (**G**) Probabilistic prediction of IL-10^+^ Treg cells (right) and IL-10^−^ Treg cells (left) in spatial transcriptomics data from CRC tumor and NAT.^45^

We next asked whether the IL-10/IL-17 axis have similar effects on human CRC tumor. Treatment of tumor organoids derived from human patients with recombinant human IL-17 accelerated tumor growth suggesting that an IL-10^+^ vs IL-10^−^ functional dichotomy observed in mice may be conserved in human CRC. To test for these subsets in human MSS CRC, we performed paired multiomic scRNA/ATAC-seq analyses of total T cell populations isolated from surgically resected primary CRC tumors and matched normal adjacent colonic tissues (NATs) from 3 patients. Analysis of total 21,676 T cells, which formed 8 distinct clusters (Figures 6A, 6B, and S7A; see Methods), showed Treg cells forming two clusters resembling mouse tumoral IL-10^+^ and IL-10^−^ Treg cells; one preferentially expressed *IL10* and *RORC*, and the other *IKZF2* gene transcripts (Figures S7B and S7C). As in mice, human IL-10^+^ Treg cells were enriched in NATs, whereas IL-10^−^ Treg cells were enriched in tumors (Figure 6C). scATAC-seq analysis using chromVar indicated similar TF motif enrichment within chromatin accessible sites between corresponding mouse and human subsets, with the enrichment of RORC motif noted in IL-10^+^ Treg cells and GATA and NR4A in IL-10^−^Treg cells (Figure 6D). To determine transcriptional similarity of human IL-10^+^ and IL-10^−^ Treg cells to their mouse counterparts, we employed mouse Treg subset-specific gene modules (Figure 3D) and determined their expression in human Treg cells. Both subsets exhibited significant enrichment for gene modules identified in their mouse counterparts (Figure 6E). Consistently, Hotspot analysis identified subset-specific gene modules in human CRC-infiltrating Treg cells corresponding to their mouse subsets. Notably, *IKZF2*, *GATA3*, *CCR8*, and *CADM1* were preferentially expressed by IL-10^−^ Treg cells, whereas *MAF* and *ZEB2* were preferentially expressed by IL-10^+^ Treg cells (Figure 6F). Together, these results demonstrated that transcriptional and chromatin states of human CRC Treg subsets resembled those of mouse IL-10^+^ and IL-10^−^ Treg cells. Notably, our analysis of published human CRC T cell datasets also identified two tumor-infiltrating Treg subsets transcriptionally similar to the ones identified by multiomic analysis (Figures S7D–S7G).^45^ The examination of the accompanying spatial transcriptomic (ST) datasets of the patients in the same cohort further corroborated the observations made in our tissue dissociation-based analysis by demonstrating an enrichment in IL-10^+^ Treg cells in NATs, while IL-10^−^ Treg cells were enriched in the tumor foci (Figures 6G, S8A–C).

Considering the opposing functions of IL-10^+^ and IL-10^−^ Treg subsets in mouse CRC, we next investigated the potential association of their relative abundance in human CRC with clinical outcomes. We leveraged bulk RNA-seq datasets of surgical CRC samples from 102 patients and deconvolved these data using published scRNA-seq datasets for total CRC cells from 10X Genomics to infer relative abundances of the two Treg subsets (see Methods).^45^ The patients were then segregated into high (H) and low (L) abundance groups based on the predicted abundance of each tumoral Treg population. Consistent with the anti-tumoral role of IL-10^+^ Treg cells established by mouse experiments, patients in the IL-10^+^ Treg high abundance group showed significantly better survival when compared to those in the corresponding low abundance group. Conversely, patients in IL-10^−^ Treg high abundance group exhibited significantly worse survival than in low abundance group, in agreement with the pro-tumoral function of this subset observed in mice (Figure S8D).

Having observed the anti-tumoral function of IL-10^+^ Treg cells and the pro-tumoral function of IL-10^−^ Treg cells in mouse CRC, and their association with better and worse human CRC prognosis, we next sought to determine whether similar populations can be identified in other human cancer types. Published single-cell atlas of T cells across 16 cancer types showed 7 distinct Treg clusters (Figure S9A).^46^ Upon closer examination, we found that clusters C0, C1, and C4 exhibited low expression for both IL-10^+^ and IL-10^−^ Treg gene modules, indicating their relatively less differentiated or activated states. Clusters C2, C3, and C5 showed high-level expression of IL-10^−^ Treg gene modules, while cluster C6 expressed a higher level of IL-10^+^ Treg signature module (Figures S9B and S9C). Although these patterns were indicative of overall transcriptional similarity of cluster 2, 3 and 5 cells to IL-10^−^ Treg cells, C3 and C5 cells were enriched only in basal cell carcinoma (BCC) and head and neck squamous cell carcinoma (HNSC), respectively, suggesting cancer type-dependent prevalence of a particular IL-10^−^ Treg flavor (Figure S9D). Importantly, both C2 and C6 populations showed significant presence in BCC, CRC, HNSC, and stomach adenocarcinoma suggesting that the dichotomy of tumoral IL-10^+^ and IL-10^−^ Treg cells is common across different barrier tissue cancers (Figure S9D).

### Treg composition and function in liver CRC metastases

**Figure 7.**
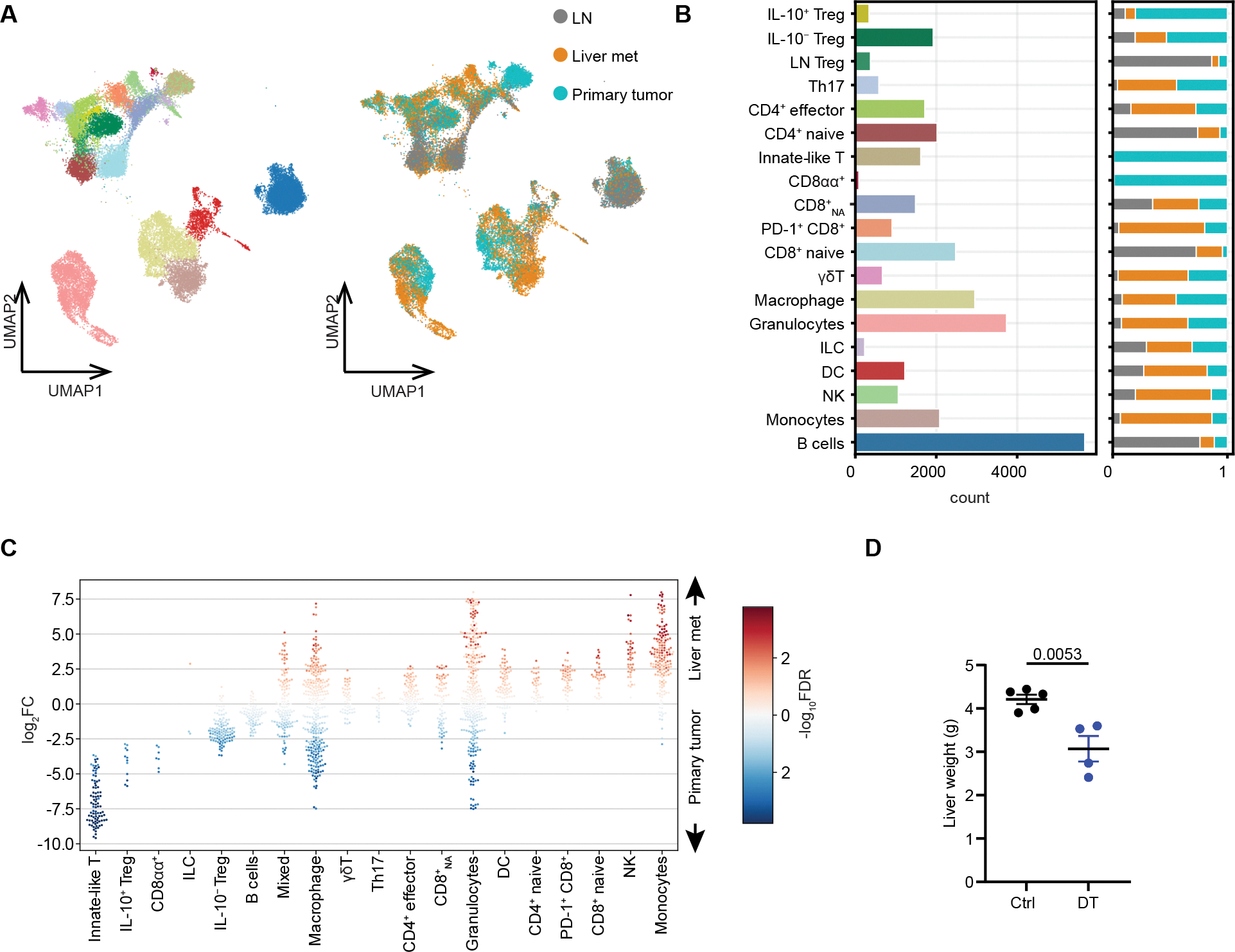
Analysis of transcriptional features and function of Treg cells in CRC liver metastases. scRNA-seq analysis of T cells isolated from primary cecal AKP tumors, cecum draining mesenteric lymph nodes, and spontaneous metastasized liver tumors 10 weeks after tumor inoculation. (**A**) UMAP plots are shown for cell type annotations (left) as indicated in (**B**) and tissue annotations (right) (n=33,218 cells). (**B**) Bar graphs depicting the number of cells for each cell type and the proportions for each tissue. (**C**) Beeswarm plots depicting log fold changes in differential abundance of cells from liver metastases versus primary tumor in neighborhoods of cells for each cell type. (**D**) Liver weights 37 days after tumor implantation. For (**D**) data were pooled from two independent experiments and are shown as mean ± s.e.m, statistical analysis was performed using unpaired *t*-tests.

Building on our findings of opposing functions of IL-10^+^ and IL-10^−^ Treg cells in primary CRC tumor, we sought to investigate whether similar dichotomy exist in liver metastases. scRNA-seq analysis of T cell populations showed predominance of IL-10^−^ Treg cells with minimal presence of IL-10^+^ Treg cells in the spontaneous liver AKP metastases contrary to the primary colonic tumors (Figures 7A and 7B). The IL-10^+^ Treg paucity was accompanied by an increased presence of macrophages, monocytes and neutrophils in the liver metastatic tumors as compared to their primary colonic counterparts, paralleling the effect of IL-10^+^ Treg depletion (Figures 7C and S5A). Nevertheless, the frequency of Th17 cells was comparable between liver and cecal tumors, likely due to the paucity of microbial antigens in the liver. Because of the overwhelming contribution of IL-10^−^ Treg cells to liver metastasis Treg population, we surmised that pan-Treg depletion would result in a reduced metastatic burden. To test this possibility, liver AKP metastases were modelled via intrasplenic injection of AKP organoids into *Foxp3*^*DTR*^ mice, which were then subjected to DT-mediated Treg ablation. Consistent with the pro-tumoral function of IL-10^−^ Treg subset and its predominance in liver AKP metastases, pan-Treg depletion resulted in a reduced liver tumor burden contrary to primary colonic AKP tumors (Figure 7D).

## DISCUSSION

CRC is a notorious outlier to the tumor-promoting function of Treg cells, with conflicting reports on how Treg abundance correlates human CRC prognosis.^7–10,21^ One potential explanation is that previous analyses did not discriminate between the contributions of *Foxp3*^hi^ Treg and *Foxp3*^lo^ effector T cells to overall *Foxp3* transcript amounts; the high numbers of the latter in CRC correlate with microbial invasion and are linked to favorable prognoses.^10^ Beyond effector T cell activation, microbiota and dietary antigens enhance colonic Treg heterogeneity, via extrathymic differentiation of RORγt^+^ (and to a lesser degree Gata3^+^) Treg cells and activation of thymic-derived Treg cells residing in the colon.^18–20^ While the majority of microbiota-specific peripheral Treg cells are RORγt^+^, only about 50% of dietary antigen induced peripheral Treg cells express RORγt.^20^ Notably, RORγt and Gata3 are enriched in IL-10^+^ and IL-10^−^ Treg subsets, respectively. It is reasonable to assume that CRC-associated microbiota, e.g., *Bacteroides fragilis*, which via polysaccharide A promotes both extrathymic Treg differentiation and IL-10 production, are shaping the dynamics of these two subsets during CRC progression.^47^

Our studies demonstrated IL-10^+^ RORγt^+^ and IL-10^−^ Helios^+^ Treg cells represent two transcriptionally and epigenetically distinct subsets residing in the mouse and human CRC TMEs and corresponding normal tissues. These subsets exhibited differential gene expression with the TCR-dependent gene signatures preferentially expressed in tumoral IL-10^−^ Treg cells but attenuated in IL-10^+^ Treg cells, consistent with TCR signaling withdrawal associated with terminal differentiation of colonic IL-10^+^ Treg cells.^21,48–51^ Most importantly, IL-10^+^ and IL-10^−^ Treg cells counteracted and promoted CRC tumor growth, respectively. This functional duality aligns with an inversion of their relative abundance in the tumors in comparison to normal adjacent tissues observed in AKP tumor-bearing mice. Notably, a similar trend was revealed by our analysis of human CRC and adjacent normal tissue ST datasets. A plausible driver is declining IL-2 as tumors grow, due to increased Treg consumption and reduced effector T cell production.

The seemingly paradoxical function of IL-10^+^ Treg cells stems from suppressing Th17-derived IL-17. Previous studies implicated IL-10 non-redundantly controls Th17 cell differentiation and function via IL-10R.^42^ Treg cells express the most IL-10 per cell among IL-10^+^ cell types and are the major IL-10-expressing T cells in the colon.^21^ Accordingly, antibody mediated IL-10R blockade and Treg-specific IL-10 ablation increased IL-17 production and tumor burden.

IL-17 exerts diverse effects on a wide range of non-transformed cell and tumor types including CRC where increased Th17 cells and IL-17A associate with worse prognosis^52,53^. Expectedly, relieving Th17 cells from IL-10^+^ Treg suppression boosted IL-17 production and increased tumor-associated neutrophils, monocytes, and macrophages, all previously linked to increased tumor burden.^34–36^ While we have not formally excluded potential contribution of these cells to enhanced AKP tumor growth, we found that unlike IL-17RA-sufficient tumors, which enlarged upon IL-10Rα blockade, IL-17RA-deficient CRC size remained unaffected despite increased IL-17. Although Th17 cells also upregulated IL-22, an epithelial maintenance factor promoting dextran sulfate sodium and azomethane-induced colon tumorigenesis,^54^ we found IL-22 signaling in AKP tumors dispensable for boosting their growth, contrary to a non-redundant role of IL-17. Together, our results suggest IL-10^+^ Treg cells decrease CRC tumor burden by suppressing Th17 cells and limiting IL-17, which promotes AKP tumor growth.

While Treg cells counteract precancerous hyperplasia and early tumor initiation by suppressing frank- or para-inflammation, they contribute to established tumor progression through suppressing tissue-damaging inflammation and providing supportive factors like amphiregulin.^1,5,55–57^ Increased Treg activity has been also linked to PD-1 blockade resistance and hyper-progression, consistent with our finding that PD-1 is preferentially expressed by pro-tumoral IL-10^+^ Treg cells.^11,58^ Previously, tumor-restraining function of IL-10 has been ascribed to rescuing exhausted CD8^+^ T cells.^59^ Our observation that IL-10^+^ Treg cells suppress the CRC-supportive factor IL-17 reveals an unexpected and previously unappreciated mechanism of Treg-mediated cancer regulation. Although our finding supports IL-10 signaling modulation as a therapeutic strategy in CRC, its pleiotropic roles complicate such an approach.^60–63^

The tumor-restraining activity of IL-10^+^ Treg cells was counterbalanced by IL-10^−^ Helios^+^ Treg cells, most likely via their superior ability to subdue tumoral CD8^+^ T cell responses. Although IL-10^−^ Treg cells also suppressed type 2 cytokines production by tumoral effector CD4^+^ T cells and consequent eosinophils recruitment, IL-4 and IL-13 neutralization did not affect CRC growth. Notably, IL-10^−^ but not IL-10^+^ Treg cells in mice and humans express high chemokine receptor CCR8, which we and others found broadly elevated on Treg cells across cancers.^4,64^ Subsequent studies revealed CCR8-depleting antibodies effectively targeted Treg cells, both as a monotherapy and combined with PD-1 or VEGF blockade or vaccination, in mouse and human cancer models.^65–67^ These results suggest that in CRC, CCR8-targeted therapy could selectively alleviate IL-10^−^ Treg cell-mediated inhibition of effector antitumoral T cell responses while maintaining IL-10^+^ Treg cell suppression of tumor-promoting factors. The functional heterogeneity observed in mice was relevant to human MSS CRC, where gene signatures IL-10^+^ Treg cells associated with favorable prognosis, while IL-10^−^ Treg cells correlated with unfavorable outcome. Furthermore, the counterparts of these subsets in other barrier tissue cancers suggest potential broader clinical relevance. The functional dichotomy offers a rationale for therapies that selectively target pro-tumoral Treg cells while sparing their anti-tumoral counterparts.

### Limitations of study

This study revealed previously unappreciated functional duality of IL-10^+^ and IL-10^−^ Treg cells in CRC. While we employed *Rorc* expression as a surrogate marker for IL-10^+^ Treg cells, the presence of minor RORγt^+^IL-10^−^ and RORγt^−^IL-10^+^ Treg subsets in both tumors and normal colon warrants their further investigation. Likewise, the specific effector mechanisms underlying tumor control accompanied by heightened Th2 and CD8^+^ T cell responses observed upon depletion of IL-10^−^ Treg cells remains to be elucidated.

## RESOURCE AVAILABILITY

### Lead Contact

Further information and requests for resources and reagents may be directed to and will be fulfilled by the lead contact, Alexander Y. Rudensky (rudenska@mskcc.org).

### Materials Availability

All reagents and resources used in this study should be directed to the lead contact. All reagents will be available based on request and completing a Materials Transfer Agreement.

### Data and Code Availability

All sequencing datasets are available on the GEO database with the following accession number: GSE290623. The single-cell Flex and VisiumHD samples were downloaded from the 10X genomics website https://www.10xgenomics.com/platforms/visium/product-family/dataset-human-crc. The pan-cancer CD4^+^ T cell atlas was downloaded from https://singlecell.mdanderson.org/TCM/. The code to reproduce the figures are available at https://github.com/snehamitra/CRC_Treg_manuscript.

## STAR METHODS

### EXPERIMENTAL MODEL ANF STUDY PARTICIPANT DETAILS

#### Mice

*Il10^tdTomato-CreER^Rosa26^lsl-YFP^Foxp3^Thy1.1^*, *Foxp3^CreER^ Il10^flox^*, *Foxp3^DTR^*, *Il10^tdTomato-Cre^ Foxp3^lsl-DTR^*, and *Foxp3^flox-DTR^* mice have been previously described.^21,68,69^ *Il10tdTomato-Cre Foxp3flox-DTR* and *Foxp3*^*CreER*^*Rosa26*^*lsl-*^YFP mice were generated by intercrossing. Co-housed littermates were used in all experiments, and all genotypes were represented in each litter analyzed. For generation of bone marrow (BM) chimeras, *Tcrb*^*tm1Mom*^ *Tcrd*^*tm1Mom*^/J mice were lethally irradiated (1000 rads) and reconstituted with 2×10^6^ T cell-depleted BM cells. The resulting chimeric mice were used for experiments after 8 weeks of BM reconstitution. All mice were housed at the Research Animal Resource Center for Memorial Sloan Kettering Cancer Center (MSKCC) and Weill Cornell Medicine. All studies were performed under the protocol 08–10-023 and approved by the Sloan Kettering Institute Institutional Animal Care and Use Committee. All animals used in this study had no previous history of experimentation and were naive at the time of analysis.

#### Human samples

A list of donor characteristics of the patients is presented in Table S1. For single-cell multiome analysis, only patients diagnosed with MSS CRC were collected. Surgical samples of tumors and adjacent normal tissues were collected from patients with prior consent to IRB protocol 06–107. All tissues were deidentified, all sex/gender and racial ethnical information were blinded and not part of the screening criteria.

#### AKP tumor organoids

The AKP tumor organoid line is a gift from Dr. Scott Lowe. The organoids were generated from female mice and maintained as previously described^22^. Briefly, organoids were amplified in 6 droplets of 40 μl of Geltrex (Corning Life Science) per well in 6-well plates, and supplemented with 3 ml of colon organoid growth media^70^ consisting of Advanced DMEM/F12 (ADF12; Thermo Fisher Scientific), 2 mM GlutaMAX (Thermo Fisher Scientific), 10 mM HEPES (Thermo Fisher Scientific), 1 mM N-acetyl-L-cysteine (Sigma-Aldrich), 1:50 B27 Supplement (Thermo Fisher Scientific), supplemented with 50 ng/mL murine EGF (Peprotech), 100 ng/mL murine Noggin (Peprotech).

#### Human CRC organoids

The human colorectal cancer (CRC) organoid line OKG136P was derived from a primary tumor and validated as previously described^71^. OKG136P was used to assess the effects of IL-17 on organoid formation and growth. Organoids were maintained in human intestinal stem cell (HISC) medium consisting of Advanced DMEM/F12 (ADF12; Thermo Fisher Scientific), 2 mM GlutaMAX (Thermo Fisher Scientific), 10 mM HEPES (Thermo Fisher Scientific), 1 mM N-acetyl-L-cysteine (Sigma-Aldrich), 1:50 B27 Supplement (Thermo Fisher Scientific), 1:100 N2 Supplement (Thermo Fisher Scientific), and 100 μg/mL Primocin (InvivoGen), supplemented with 50 ng/mL EGF (Peprotech), 100 ng/mL Noggin (Peprotech), 500 nM A83–01 (Sigma-Aldrich), 50 ng/mL FGF2 (Peprotech), and 100 ng/mL IGF-I (Peprotech). The mutational profile of OKG136P includes TP53(R248W), PIK3R3(L181Tfs13), APC(W553), SOX9(S414Lfs164), PIK3R1(I484Mfs4), SF3B1(R702Q), JUN(S267F), and SOX9(L81_W86dup).

### METHOD DETAILS

#### AKP tumor inoculation

AKP tumor organoid were maintained as previously described.^22^ For each primary tumor experiment, tumor organoid 3D culture was digested with TrypLE and resuspend in PBS:Geltrex (2:1) mixture at the density of 10–20 × 10^6^ cells/ml and maintained on ice. 20ul of the cell suspension was injected into the subserosa space of mouse cecum. For intra-splenic injection model of liver metastasis, mice were anesthetized and placed in the right lateral decubitus position. The left flank was shaved and disinfected, and a left subcostal incision (~1–1.5 cm) was made to expose and exteriorize the spleen. The spleen was ligated at the midpoint using two sterile absorbable sutures and divided between the ligatures with surgical scissors. A 100 μL suspension of tumor organoids in PBS (4×10^5 cells) was injected into the proximal splenic tissue using a 31G insulin syringe. The injected spleen is then excised to prevent local tumor growth after ligation of its vascular supply. The peritoneal wall was closed with absorbable suture, and the skin was closed using clips.

#### Tumor organoids *in vitro* cytokine treatment

AKP tumor organoid were cultured *in vitro* as previously described.^22^ On day 1, cells were seeded at 0.67 million cells/ml in PBS:Geltrex (1:2) mixture and plated into 40 ml droplets at 1 drop per well concentration in a 24 well plate. Cells were treated with IL-17A and IL-22 at a concentration of 50 ng/ml each on day 1 and day 3. Cells from each well are harvested and counted on day 7.

For human tumor organoid formation assay, live cells were isolated from day 7 cultures of OKG136P, and 1,000 cells were embedded in 20 μL Matrigel domes per well in 24-well suspension culture plates. Organoids were cultured in HISC medium with or without IL-17 (STEMCELL Technologies, #78032.1). To assess organoid growth, Z-stack images were acquired using a BioTek Cytation5 plate reader at day 7 after single cell plating, and maximum intensity projections were generated for each well. Quantification was performed using the cellular analysis module of the Cytation5 software.

#### Gene editing of AKP tumor organoid

AKP organoids were electroporated using previously described protocol.^72^ 2 μg of *in vitro* transcribed Cas9 mRNA, 200 pmol of sgRNAs (5’: GTGTTGATTACCGATGAGAA; 3’: GCAGCTAATCACCCCTAAGG) each, and 2 μg of linearized donor plasmid were used per electroporation. Donor plasmids were designed with 40bp homology arms flanking both sgRNA cut sites. A targeting construct consisted of a EF1α promoter driving the Thy1.1 coding sequence, followed by a STOP codon, and polyA sequence was inserted into the *Il17ra* locus by microhomology-mediated end joining. The success of the knock-in was verified with genotyping PCR (Il17ra-F: GACTACTTGGCAGCAGAGCA; Il17ra-R: GCCAAGCCAGCACCACTG; EF1a-R: ACTTTCCCAGTTTACCCCGC) and flow cytometric analysis of Thy1.1 reporter expression. Organoid cells were FACS sorted based on their Thy1.1 expression twice before inoculation. For control organoid, only Cas9 RNA was used for the electroporation process.

#### Mouse treatments

For diphtheria toxin (DT) treatment, DT was reconstituted in sterile PBS at 1 mg/ml and frozen at −80°C in single use aliquots. Aliquots were thawed and diluted 200 x in PBS. For inactivated control (bDT), this 1 ml dilution was heated at 95–100°C for 30 minutes. Both active and control DT were filtered through 0.22 μm syringe-driven filters. For primary tumor experiments, mice were injected intravenously with 200 μl of this dilution for the first doses (1000 ng DT) and 200 μl of a 1:1 dilution with PBS for the second dose (500 ng DT) or intraperitoneally with 200 μl of a 1:1 dilution with PBS for the second dose for subsequent doses (500 ng DT). For liver metastasis experiments, mice were injected intraperitoneally with 1000 ng of DT for the first dose and with 500ng of DT for all subsequent doses. For antibody treatment in tumor-bearing mice, the first dose immediately after the surgery was given by retroorbital i.v. injection, while all the subsequent injections were given i.p. PD1 antibody was injected on day 8, 11, 14, 17, and 20 at a dosage of 0.25 mg/mouse in 200 ml of saline. IL-10Rα antibody was injected on day 1 and day 8 after surgery at a dosage of 1 mg/mouse in 100 ml of saline. IL-4 and anti-IL-13 antibodies were injected on day 1, 4, 7, 10, and 13 at a dosage of 50 μg/mouse each in 200ul of saline as previously described.^73^ For tamoxifen-induced Cre-loxP mediated gene recombination, mice were gavaged with 8 mg tamoxifen dissolved in 200 μl corn oil (Sigma-Aldrich). Tamoxifen was dissolved by gentle agitation at 37 °C overnight. Aliquots were frozen (−80 °C) and thawed as needed throughout the experiments. For each experiment, mice were gavaged twice 9 and 6 days before tumor transplantation.

#### Cell isolation for flow cytometry

Mice were injected retro-orbitally with 1.5 μg anti-mouse CD45.2 (Brilliant Violet 510 conjugated, BioLegend 109838) in 200 μl sterile PBS 3 min prior to euthanasia to label and exclude blood-exposed cells. All centrifugations were performed at 700 x g for 3 min at 4 °C. Secondary lymphoid organs were dissected and placed in 1 ml wash medium (RPMI 1640, 2% Fetal Bovine Serum (FBS), 10 mM HEPES buffer, 1% penicillin/streptomycin, 2 mM L-glutamine). Tissues were then mechanically disrupted with the back end of a syringe plunger, and then passed through a 100 μm, 44% open area nylon mesh. For tumor and cecum, the cecal pouch was dissected, and after removal of fat and the cecal patch, opened longitudinally and vigorously shaken in 1x PBS to remove luminal contents. Tumor was then dissected from the surrounding cecal tissue. Both tumor and cecum were placed in a 50 ml screw-cap tube with 25 ml wash medium supplemented with 5 mM EDTA and 1 mM dithiothreitol and shaken horizontally at 250 RPM for 15 to 20 min at 37°C. After a 5 sec vortexing, epithelial and immune cells from the epithelial layer were removed by filtering the suspension through a tea strainer. Remaining tissue was placed back in 50 ml tubes, washed with 25 ml wash medium, strained again, and replaced in 5 ml tubes. Tumor was minced into pieces with the size of approximately 1–2 mm diameters. 25 ml wash medium supplemented with 0.2 U/ml collagenase A, 4.8 mM calcium chloride, 1 U/ml DNase I was added, along with four ¾ inch ceramic beads, and tissues were shaken horizontally at 250 RPM for 35 min at 37°C. Suspension was then passed through a 100 μm strainer, centrifuged to remove debris and collagenase solution, and then washed by centrifugation in 40% Percoll^™^ in wash medium. All enzymatically digested samples were washed by centrifugation in 5 ml wash medium.

#### Flow cytometry

To assess cytokine production after ex vivo restimulation, single cell suspensions were incubated for 4 hours at 37°C with 5% CO2 in the presence of 50 ng/ml PMA and 500 ng/ml ionomycin with 1 μg/ml brefeldin A and 2 μM monensin to inhibit ER and Golgi transport. For flow cytometric analysis, cells were stained in 96 well V-bottom plates. All centrifugations were performed at 700x g for 4 min at 4 °C for live cells and 900x g for 2 min for fixed cells. Staining with primary antibodies was carried out in 100 μl for 25 min at 4°C in staining buffer (PBS, 0.2 % (w/v) BSA, 2 mM EDTA, 10 mM HEPES, 0.1% (w/v) NaN3). Cells were then washed with 200 μl PBS and then concurrently stained with Zombie NIR^™^ Fixable Viability dye for 10 min at room temperature. Cells were washed with 100 μl staining buffer, resuspended in 200 μl staining buffer, and passed through a 100 μm nylon mesh. For cytokine staining, cells were fixed and permeabilized with BD Cytofix/Cytoperm per manufacturer instructions. Intracellular antigens were stained overnight hour at 4°C in the 1x Perm/Wash buffer. Samples were then washed twice in 200 μl 1x Perm/Wash buffer, resuspending each time, resuspended in 200 μl staining buffer, and passed through a 100 μm nylon mesh. All samples were acquired on an Aurora cytometer (Cytek Biosciences) and analyzed using FlowJo v10 (BD Biosciences).

#### Cell sorting

For mouse samples, cell isolation was performed as described above, except that mice were perfused, and samples were not washed with 40% Percoll^™^. Staining was performed as described above, except buffer contained 2 mM L-glutamine and did not contain NaN3, with staining volume adjusted to 500 μl and washes adjusted to 5 ml and staining performed in 15 ml screw-cap tubes. 1 μg ‘HashTag’ antibodies (BioLegend) were added to extracellular antigen stain for scRNA-seq sorting, and scRNA-seq sort samples were not DNase I treated. Samples were resuspended in Wash buffer supplemented 5mM EDTA for sorting and sorted into wash medium in 1.5 ml Protein LoBind tubes. For human CRC tissues, samples were weighed and dissociated with Tumor Dissociation Kit, Human (Miltenyi Biotec) on a gentleMACS^™^ dissociator (Miltenyi Biotec) using programs recommended for CRC samples. Suspension was then passed through a 100 μm strainer, centrifuged to remove debris and collagenase solution, and then washed by centrifugation in RPMI extensively.

All sorting was performed on an Aria II (BD Biosciences) or an AuroraCS cytometer (Cytek Biosciences).

#### Histological analysis

The entire tumor tissue with ~0.5 cm of surrounding cecal tissue were fixed in 4% PFA for > 48 hours. Tissue embedding, sectioning, and staining were carried out by the Molecular Cytology Core at MSKCC.

#### Single-cell sequencing data preprocessing

The FASTQ files from all the single-cell datasets were aligned using the Cell Ranger pipeline. We performed quality control and applied different filtering thresholds for each dataset. For the mouse scRNA-seq data from the single-cell multiome of RNA+ATAC at 4 weeks, we restricted the percentage of mitochondrial reads (pct_counts_mt) to less than 10%, the number of genes expressed in each cell (n_genes_by_counts) to a maximum of 3,000, and the UMI counts (total_counts) to at least 1,000. For the scATAC-seq data from the same multiome, we retained cells with at least 180 fragments and a TSS enrichment score of at least 3. The TSS enrichment score is the ratio of the number of reads at the gene TSS relative to the flanking regions.^74^ For the mouse scRNA-seq data from 2, 4, and 6 weeks, we retained cells with pct_counts_mt less than 20%, n_genes_by_counts at most 3,000, and total_counts at least 500. We generated scRNA-seq data sets separately for the T cells and other immune cells from IL-10 depleted tumors and control tumors. For the scRNA-seq of T cells we retained cells with n_genes_by_counts at least 1,000 and at most 4,000, total_counts at most 12,000, and pct_counts_mt less than 10%. For the non T cells we retained cells with n_genes_by_counts at most 2,500, total_counts at most 8,000, and pct_counts_mt less than 20%. In case of the metastasis scRNA-seq data from 10 weeks, we retained cells with pct_counts_mt less than 7%, n_genes_by_counts less than 4,200, and total_counts greater than 500. Finally, for the scRNA-seq from the multiome of patient samples, we extracted cells with pct_counts_mt less than 20%, n_genes_by_counts greater than 800, and total_counts less than 6,000. The matched scATAC-seq data from the patient samples were filtered to retain cells with at least 1,000 fragments and a TSS enrichment score of at least 4. For the multiome data sets, we retained the cells that passed quality control (QC) thresholds in both scRNA-seq and scATAC-seq.

#### Batch correction and cell-type annotation

The single-cell multiome of the patient samples had strong batch effects in both scRNA-seq and scATAC-seq. To this end, we used combat^75,76^ on the log-normalized counts from scRNA-seq to generate batch-corrected counts. Using the batch-corrected counts, we performed principal component analysis (PCA) that we used to generate a UMAP embedding for visualization. We ran CellSpace^77^ on the top 25,000 variably accessible 500bp tiles with the margin parameter set to 0.1 to generate a batch corrected latent space embedding of the scATAC-seq data. This latent space embedding was used to generate a UMAP embedding for visualization. All the other remaining data sets did not have batch effects. In those cases, we performed dimensionality reduction using PCA on the normalized scRNA-seq counts and iterative LSI^74^ on the scATAC-seq tiles followed by UMAP generation. We performed Leiden clustering on the PCA components of scRNA-seq for all the data sets. The clustering was performed in an iterative manner where we sub-clustered specific clusters to obtain clearer signal of specific phenotypes. The cluster labels were transferred to scATAC-seq data for chromatin accessibility-based analyses.

#### Transcription factor binding activity analysis

Peaks were called in the scATAC-seq data sets using Macs2^78^ by grouping the cells into pseudo-bulks based on the cell-type annotations obtained from matched scRNA-seq. chromVAR^79^ was run on these peak sets to quantify the enrichment of TF binding sites in the accessible regions of individual cells.

#### Prediction of regulatory regions from single-cell multiome

SCARlink^27^ was run on the subsets of CD4^+^ T cells from the mouse single-cell multiome. We trained models on 4,315 genes that were highly variable with detectable transcripts in at least 10% of all the CD4^+^ T cells and extracted the predicted gene-linked enhancer tiles with z-score greater than 0.5 and false discovery rate (FDR) of 0.05 to compare the number of gene-specific enhancers predicted for IL-10^+^ and IL-10^−^ Tregs.

#### Identification of gene modules

Hotspot^28^ was used separately on mouse and human scRNA-seq data to identify gene modules for Tregs. First, cells that were not Treg cells were filtered out alongside with genes that were not expressed in any of the Treg cells. Malat1/MALAT1, mitochondrial genes, and ribosomal genes were also filtered out. k-nearest neighbor (kNN) graphs were generated with 30 neighboring cells. For the mouse data, we generated modules with at least 25 genes at FDR < 0.05 and with at least 35 genes at FDR < 0.0001 for human data. This gave us seven gene modules in the mouse data and five gene modules in the human data. The gene scores calculated using these gene modules were generated using the score genes() function from scanpy.^76^

#### Analysis of single-cell flex and VisiumHD 10x Genomics datasets

Cell Ranger processed output was first extracted to retain cells with mitochondrial read percentage of less than 25% and UMI counts greater than 500. Then we log-normalized the count matrix and performed PCA on the top 2,000 highly variable genes. We performed Leiden clustering to obtain the broad cell type categories. We then extracted the T cells and re-clustered the cells to identify granular cell populations.

We analyzed the VisiumHD data sets at 8 μm resolution. We first filtered and retained spots with mitochondrial reads percentage less than 25%, and UMI counts greater than 500. We then used Tacco^80^ to annotate the cell types in the Visium HD samples using the single-cell Flex data as reference. Since we have matched samples in both Flex and Visium, we used the identifical sample in Flex as reference, when performing the label transfer. We used the annotate() function in TACCO with parameters multi_center=3 and lamb=1e-3.

#### Differential neighborhood abundance analysis

We used Milo^81^ to test the differential abundance of immune cells from primary tumor versus immune cells from liver metastases. To this end, we constructed a kNN graph over 25 neighbors to aggregate cells into neighborhoods. We performed differential abundance testing over these neighborhoods having three replicates from primary tumor and four replicates from liver metastases. We then annotated the individual neighborhoods based on the cell types forming 60% majority each neighborhood. If a given neighborhood did not comprise of a cell type forming 60% majority, we annotated those as ‘mixed’ neighborhoods.

#### Survival analysis

We ran BayesPrism^82^ to deconvolve the bulk RNA-seq data sets using the 10x Genomics single-cell flex as reference. This generated probabilistic predictions of deconvolution for each cell type in the reference atlas. We applied quantile cutoffs on the probabilistic predictions of IL-10^+^ and IL-10^−^ Treg cells and generated Kaplan-Meier survival plots.

### QUANTIFICATION AND STATISTICAL ANALYSIS

For flow cytometric analysis and tumor experiments, all statistical analyses were performed using Prism (GraphPad software). Data were analyzed using unpaired *t* test, one-way ANOVA or two-way ANOVA as indicated in figure legends and are presented as mean ± s.e.m. *p* values are shown in the plots. For sequencing data analysis, all gene score comparisons were performed using Wilcoxon rank-sum test. All differential gene expression volcano plots and cell frequency analyses were performed using unpaired *t* tests with Benjamini-Hochberg correction for *p* values. The survival analyses were performed using Kaplan-Meier test.

## Supplementary Material

Supplementary Material

## Figures and Tables

**KEY RESOURCE TABLE:** 

REAGENT or RESOURCE	SOURCE	IDENTIFIER
Antibodies		
Anti-mouse Ki-67 Monoclonal Antibody (SolA15), Alexa Fluor^™^ 532	Thermo Fisher Scientific	Cat# 58-5698-82, RRID:AB_2802365
Anti-mouse Bcl-6 Monoclonal Antibody (K112-91), Alexa Fluor^™^ 647	BD Biosciences	Cat# 561525, RRID:AB_10898007
Anti-mouse Siglec H Monoclonal Antibody (551), Alexa Fluor^™^ 647	BioLegend	Cat# 129607, RRID:AB_2189146
Anti-mouse CD90.2 Monoclonal Antibody (30-H12), Alexa Fluor^™^ 700	BioLegend	Cat# 105320, RRID:AB_493725
Anti-mouse IL-4 Monoclonal Antibody (11B11), APC	Thermo Fisher Scientific	Cat# 17-7041-82, RRID:AB_469494
Anti-mouse NK1.1 Monoclonal Antibody (PK136), APC-eFluor^™^ 780	Thermo Fisher Scientific	Cat# 47-5941-82, RRID:AB_2735070
Anti-mouse CD86 (B7-2) Monoclonal Antibody (GL1), APC-eFluor^™^ 780	Thermo Fisher Scientific	Cat# 47-0862-82, RRID:AB_2815162
Anti-mouse CD170 (Siglec-F) Monoclonal Antibody (S17007L), Biotinylated	BioLegend	Cat# 155512, RRID:AB_2814066
Anti-mouse CD44 Monoclonal Antibody (IM7), Brilliant Blue 700	BD Biosciences	Cat# 566506, RRID:AB_2744396
Anti-mouse Ly-6G Monoclonal Antibody (1A8), Brilliant Blue 700	BD Biosciences	Cat# 566435, RRID:AB_2739730
Anti-mouse CD25 Monoclonal Antibody (PC61), Brilliant UltraViolet 395	BD Biosciences	Cat# 564022, RRID:AB_2722574
Anti-mouse CD11b Monoclonal Antibody (M1/70), Brilliant UltraViolet 395	BD Biosciences	Cat# 563553, RRID:AB_2738276
Anti-mouse CD19 Monoclonal Antibody (1D3), Brilliant UltraViolet 395	BD Biosciences	Cat# 563557, RRID:AB_2722495
Anti-mouse CD4 Monoclonal Antibody (RM4-5), Brilliant UltraViolet 496	Thermo Fisher Scientific	Cat# 364-0042-82, RRID:AB_2920954
Anti-mouse CD45R (B220) Monoclonal Antibody (RA3-6B2), Brilliant UltraViolet 496	BD Biosciences	Cat# 564662, RRID:AB_2722578
Anti-mouse CD62L Monoclonal Antibody (MEL-14), Brilliant UltraViolet 563	BD Biosciences	Cat# 741230, RRID:AB_2870784
Anti-mouse CD117(c-kit) Monoclonal Antibody (2B8), Brilliant UltraViolet 563	Thermo Fisher Scientific	Cat# 365-1171-80, RRID:AB_2925386
Anti-mouse CD274, PD-L1 Monoclonal Antibody (MIH5), Brilliant UltraViolet 615	BD Biosciences	Cat# 752339, RRID:AB_2875856
Anti-mouse γδTCR Monoclonal Antibody (GL3), Brilliant UltraViolet 661	BD Biosciences	Cat# 750410, RRID:AB_2874580
Anti-mouse FceR1a Monoclonal Antibody (MAR-1), Brilliant UltraViolet 661	BD Biosciences	Cat# 751765, RRID:AB_2875742
Anti-mouse CD8α Monoclonal Antibody (53-6.7), Brilliant UltraViolet 737	BD Biosciences	Cat# 564297, RRID:AB_2722580
Anti-mouse CD80 (B7-1) Monoclonal Antibody (16-10A1), Brilliant UltraViolet 737	BD Biosciences	Cat# 612773, RRID:AB_2870102
Anti-mouse TCRβ Monoclonal Antibody (H57-597), Brilliant UltraViolet 805	BD Biosciences	Cat# 748405, RRID:AB_2872824
Anti-mouse CD127 (IL-7Rα) Monoclonal Antibody (A7R34), Brilliant Violet 421	BioLegend	Cat# 135023, RRID:AB_10897948
Anti-mouse CD197 (CCR7) Monoclonal Antibody (4B12), Brilliant Violet 421	BioLegend	Cat# 120120, RRID:AB_2561446
Anti-mouse IL-10 Monoclonal Antibody (JES5-16E3), Brilliant Violet 421	BioLegend	Cat# 505021, RRID:AB_10900417
Anti-mouse CD103 Monoclonal Antibody (M290), Brilliant Violet 480	BD Biosciences	Cat# 566118, RRID:AB_2739520
Anti-mouse MHC Class II Monoclonal Antibody (M5/114.15.2), Brilliant Violet 480	BD Biosciences	Cat# 566086, RRID:AB_2869739
Anti-mouse CD45 Monoclonal Antibody (30-F11), Brilliant Violet 570	BioLegend	Cat# 103136, RRID:AB_2562612
Streptavidin, Brilliant Violet 570	BioLegend	Cat# 405227, RRID:N/A (streptavidin)
Anti-mouse CD152 (CTLA4, CTLA-4, Ly-56) Monoclonal Antibody (UC10-4B9), Brilliant Violet 605	BioLegend	Cat# 106323, RRID:AB_2566467
Anti-mouse CD64 (FcγRI) Monoclonal Antibody (X54-5/7.1), Brilliant Violet 605	BioLegend	Cat# 139323, RRID:AB_2629778
Anti-mouse CD3e Monoclonal Antibody (17A2), Brilliant Violet 605	BioLegend	Cat# 100237, RRID:AB_2562039
Anti-mouse CXCR3 (CD183) Monoclonal Antibody (CXCR3-173), Brilliant Violet 650	BioLegend	Cat# 126531, RRID:AB_2563160
Anti-mouse XCR1 Monoclonal Antibody (ZET), Brilliant Violet 650	BioLegend	Cat# 148220, RRID:AB_2566410
Anti-mouse CD44 Monoclonal Antibody (IM7), Brilliant Violet 650	BioLegend	Cat# 103049, RRID:AB_2562600
Anti-mouse KLRG1 Monoclonal Antibody (2F1), Brilliant Violet 711	BD Biosciences	Cat# 564014, RRID:AB_2738542
Anti-mouse Ly6C Monoclonal Antibody (HK1.4), Brilliant Violet 711	BioLegend	Cat# 128037, RRID:AB_2562874
Anti-mouse IFN-γ Monoclonal Antibody (XMG1.2), Brilliant Violet 711	BioLegend	Cat# 505836, RRID:AB_2565494
Anti-mouse CD11b Monoclonal Antibody (M1/70), Brilliant Violet 750	BioLegend	Cat# 101267, RRID:AB_2810328
Anti-mouse CD45 Monoclonal Antibody (30-F11), Brilliant Violet 750	BioLegend	Cat# 103157, RRID:AB_2734155
Anti-mouse CD279 (PD1, PD-1) Monoclonal Antibody (29F.1A12), Brilliant Violet 785	BioLegend	Cat# 135225, RRID:AB_2563680
Anti-mouse CX3CR1 Monoclonal Antibody (SA011F11), Brilliant Violet 785	BioLegend	Cat# 149029, RRID:AB_2565938
Anti-mouse CD90.2 Monoclonal Antibody (53-2.1), Brilliant Violet 786	BD Biosciences	Cat# 564365, RRID:AB_2734760
Anti-mouse Gata-3 Monoclonal Antibody (TWAJ), eFluor 450	Thermo Fisher Scientific	Cat# 48-9966-42, RRID:AB_2811834
Anti-mouse CD317 Monoclonal Antibody (eBio927), eFluor 450	Thermo Fisher Scientific	Cat# 48-3172-80, RRID:AB_2043880
Anti-mouse IL-17a Monoclonal Antibody (eBio17B7), eFluor 450	Thermo Fisher Scientific	Cat# 48-7177-80, RRID:AB_11149677
Anti-mouse Foxp3 Monoclonal Antibody (FJK-16s), FITC	Thermo Fisher Scientific	Cat# 11-5773-82, RRID:AB_465243
Anti-mouse T-Bet Monoclonal Antibody (4B10), PE	Thermo Fisher Scientific	Cat# 12-5825-82, RRID:AB_925761
Anti-mouse RORγt Monoclonal Antibody (Q31-378), PE-CF594	BD Biosciences	Cat# 562684, RRID:AB_2651150
Anti-mouse CD45R (B220) Monoclonal Antibody (RA3-6B2), PE-Cy5	Tonbo Biosciences	Cat# 55-0452, RRID:AB_2621821
Anti-mouse NK1.1 Monoclonal Antibody (PK136), PE-Cy5	BioLegend	Cat# 108716, RRID:AB_493590
Anti-mouse IL-2 Monoclonal Antibody (JES6-5H4), PE-Cy5	BioLegend	Cat# 503824, RRID:AB_2123674
Anti-mouse CD19 Monoclonal Antibody (1D3), PE-Cy5.5	Thermo Fisher Scientific	Cat# 35-0193-82, RRID:AB_891395
Anti-mouse CD185 (CXCR5) Monoclonal Antibody (L138D7), PE-Cy7	BioLegend	Cat# 145516, RRID:AB_2562210
Anti-mouse Foxp3 Monoclonal Antibody (FJK-16s), PE-Cy7	Thermo Fisher Scientific	Cat# 25-5773-82, RRID:AB_891552
Anti-mouse CD11c Monoclonal Antibody (N418), PE-eFluor610	Thermo Fisher Scientific	Cat# 61-0114-82, RRID:AB_2574530
Anti-mouse IL-13 Monoclonal Antibody (eBio13A), PE-eFluor610	Thermo Fisher Scientific	Cat# 61-7133-82, RRID:AB_2574654
Anti-mouse CD39 Monoclonal Antibody (24DMS1), PerCP-eFluor 710	Thermo Fisher Scientific	Cat# 46-0391-82, RRID:AB_10717953
Anti-mouse IL-22 Monoclonal Antibody (1H8PWSR), PerCP-eFluor 710	Thermo Fisher Scientific	Cat# 46-7221-80, RRID:AB_1944468
Anti-mouse MHC Class II Monoclonal Antibody (M5/114.15.2), Spark Blue 550	BioLegend	Cat# 107662, RRID:AB_2860616
Anti-human CD3 Monoclonal Antibody (OKT3), Brilliant Violet 605	BioLegend	Cat# 317321, RRID:AB_11126166
Anti-human CD4 Monoclonal Antibody (OKT4), FITC	BioLegend	Cat# 317408, RRID:AB_571951
Anti-human CD8α Monoclonal Antibody (HIT8a), PerCP-Cy5.5	BioLegend	Cat# 300924, RRID:AB_1575074
Anti-human CD45 Monoclonal Antibody (HI30), PE-Dazzle594	BioLegend	Cat# 304052, RRID:AB_2563568
Anti-human CD25 Monoclonal Antibody (BC96), PE-Cy7	Thermo Fisher Scientific	Cat# 25-0259-42, RRID:AB_1257140
Biological samples		
CRC Patient tumor and adjacent normal tissue samples	MSKCC	N/A
Chemicals, peptides, and recombinant proteins		
Brefeldin A	Millipore Sigma	Cat# B7651
Collagenase A from *Clostridium histolyticum*	Sigma-Aldrich	Cat# 11088793001
Diphtheria Toxin	List Biological Laboratories	Cat# 150
DNase I grade II, from bovine pancreas	Sigma-Aldrich	Cat# 10104159001
Ionomycin calcium salt	Millipore Sigma	Cat#I0634
Monensin sodium salt	Millipore Sigma	Cat# M5273
Phorbol 12-myristate 13-acetate (PMA)	Millipore Sigma	Cat# P8139
Tamoxifen	Sigma-Aldrich	Cat# T5648-5G
Geltrex	Corning Life Science	Cat# A1413302
TrypLE Express Enzyme	Thermo Fisher Scientific	Cat# 12604013
Advanced DMEM/F-12	Thermo Fisher Scientific	Cat# 12634010
GlutaMAX Supplement	Thermo Fisher Scientific	Cat# 35050061
GlutaminePlus - 200mM	Fisher Scientific	Cat# MT25005CI
MEM Nonessential Amino Acids 100 mM	Fisher Scientific	Cat# MT25025CI
Sodium Pyruvate	Fisher Scientific	Cat# MT25000CI
Pen-Strep Solution	Fisher Scientific	Cat# MT30002CI
HEPES buffer	Fisher Scientific	Cat# 25-060-CI
B-27 Supplement	Thermo Fisher Scientific	Cat# 17504044
N-Acetyl-L-Cysteine	Sigma-Aldrich	Cat# A9165-5G
Mouse Recombinant EGF Protein	Thermo Scientific	Cat# PMG8041
Mouse Recombinant Noggin	PeproTech	Cat# 250-38-500UG
N-2 Supplement	Thermo Fisher Scientific	Cat# 17502048
Primocin	Invivogen	Cat# ant-pm-2
Human Recombinant EGF Protein	Thermo Fisher Scientific	Cat# AF-100-15-500UG
Human Recombinant Noggin	Thermo Fisher Scientific	Cat# 120-10C-20UG
A 83-01	Thermo Fisher Scientific	Cat# SML0788-5MG
Human FGF-basic (FGF-2/bFGF) (154 aa)	Thermo Fisher Scientific	Cat # 100-18B-50UG
Human IGF-I Recombinant Protein	Thermo Fisher Scientific	Cat# 100-11-500UG
Human Recombinant IL-17A	Stemcell	Cat# 78032
Mouse Recombinant IL-4	PeproTech	Cat# 214-14-20UG
Mouse Recombinant IL-13	Thermo Fisher Scientific	Cat# 210-13-10UG
Mouse Recombinant IL-17A	PeproTech	Cat# 210-17-5UG
Corning Matrigel, GFR, LDEV-Free	Corning Life Science	Cat# 354230
Zombie NIR LiveDead	BioLegend	Cat# 423106
SYTOX^®^ Blue Dead Cell Stain	Thermo Fisher Scientific	Cat# S34857
Critical commercial assays		
Chromium Next GEM Single Cell 5’ Kit v2	10x Genomics	Cat# 1000263
Chromium Single Cell 3’ GEM, Library & Gel Bead Kit v3	10x Genomics	Cat# 1000075
Chromium Single Cell Mouse TCR Amplification Kit	10x Genomics	Cat# 1000254
eBioscience Foxp3/Transcription Factor Staining Buffer Set	ThermoFisher	Cat# 00-5523-00
Fixation/Permeabilization Solution Kit (Cytofix/Cytoperm)	BD Biosciences	Cat# 554714
BD Transcription Factor Buffer Set	BD Biosciences	Cat# 562574
Deposited data		
Mouse CRC multiome data	This study	GEO: GSE290623
Mouse CRC time course data	This study	GEO: GSE290623
IL-10^+^ Treg cell depleted CRC sequencing data	This study	GEO: GSE290623
Human CRC multiome data	This study	GEO: GSE290623
Human CRC bulk RNA-seq data	This study	GEO: GSE290623
Human CRC single cell and spatial transcriptomics data	PMID: 40473992	GEO: GSE280318
Pan-cancer CD4 T cell atlas data	PMID: 37248301	https://singlecell.mdanderson.org/TCM/
Experimental models: Cell lines		
AKP organoid	Lowe Lab	N/A
OKG136P organoid	Ganesh Lab	N/A
Experimental models: Organisms/strains		
Mouse: *Il10^tdTomato-CreER^*	PMID: 39905200	N/A
Mouse: *Il10^tdTomato-Cre^*	PMID: 39905200	N/A
Mouse: *Foxp3^LSL-DTR^*	PMID: 39905200	N/A
Mouse: *Foxp3^flox-DTR^*	PMID: 28607488	MGI:6758784
Mouse: *Foxp3^Thy1.1^*	PMID: 18695219	MGI:5451201
Mouse: *Rosa26^lsl-YFP^*	PMID: 11299042	MGI:2449038
Mouse: *Foxp3^CreER^*	PMID: 20929851	JAX:016961
Mouse: *Il10^flox^*	PMID: 15534372	JAX:036598
Mouse: *Foxp3^DTR^*	PMID: 17136045	JAX:016958
Mouse: B6.129P2-*Tcrb^tm1Mom^ Tcrd^tm1Mom^*/J	PMID: 1359428	JAX: 002122
Mouse: C57BL/6J	The Jackson Laboratory	JAX:000664
Oligonucleotides		
sgRNA targeting *Il17ra*	This study	N/A
Genotyping primer for *Il17ra*	This study	N/A
*in vitro* transcribed Cas9 mRNA	Ganesh Lab	N/A
Recombinant DNA		
Donor DNA for *Il17ra *KIKO	Twist Biosciences	Cat# Q-387523
Software and algorithms		
GraphPad Prism 10.0	GraphPad	https://www.graphpad.com/scientific-software/prism/
FlowJo v10	Tree Star	https://www.flowjo.com/solutions/flowjo
Biorender	Biorender	www.biorender.com
Scanpy (version 1.11.4)	Wolf *et al.*^76^	https://scanpy.readthedocs.io/en/stable/
ArchR (version 1.0.3)	Granja *et al.*^74^	https://www.archrproject.com/
chromVAR (version 1.20.0)	Schep *et al.*^79^	https://greenleaflab.github.io/chromVAR/
Seurat (version 5.0.2)	Hao *et al.*^83^	https://satijalab.org/seurat/
Hotspot (version 1.1.1)	DeTomaso *et al.*^28^	https://hotspot.readthedocs.io/
SCARlink (version 1.0.0)	Mitra *et al.*^27^	https://github.com/snehamitra/SCARlink
Pertpy (0.7.0)	Heumos et al.^84^	https://pertpy.readthedocs.io/
scipy (version 1.16.2)	Virtanen *et al.*^85^	https://scipy.org/
Tacco (version 0.4.0.post1)	Mages *et al.*^80^	https://simonwm.github.io/tacco/
msigdbr (version 25.1.1)	Dolgalev	https://igordot.github.io/msigdbr
ClusterProfiler (version 4.10)	Yu *et al.*^86^	https://www.bioconductor.org/packages/release/bioc/vignettes/clusterProfiler/inst/doc/clusterProfiler.html
BayesPrism (version 2.2.2)	Chu *et al.*^82^	https://github.com/Danko-Lab/BayesPrism
survival (version 3.5.8)	CRAN	https://github.com/therneau/survival
Analysis	This paper	https://github.com/snehamitra/CRC_Treg_manuscript
Other		
Cytek Aurora System	Cytek	N/A
Cytek Aurora CS	Cytek	N/A

## Highlights:

- IL-10^+^ and IL-10^−^ Treg cells constitute two functionally distinct subsets in CRC
- IL-10^+^ Treg cells restrain tumor growth by suppressing IL-17 production by CD4^+^ T cells
- IL-10^+^ and IL-10^−^ Treg cell abundances correlate with opposite prognoses in human CRC
