# Supplementary material for: Opposing Functions of Distinct Regulatory T Cell Subsets in Colorectal Cancer
Source: Immunity. Author manuscript; Available in PMC 2026 Feb 12. (PMC12895308; doi:10.1016/j.immuni.2025.11.014)
Supplement: Supplementary Material [file NIHMS2129381-supplement-Supplementary_Material.pdf]

Figure S1. Distinct immune cell composition of human MMRd and MMRp CRC tumors, related to Figure 1.

Re-analysis of published scRNA-seq CRC datasets.[24] Fraction of Treg cells and macrophages and differential *GZMB* and *IFNG* expression across patient samples grouped by MMRd and MMRp CRCs.

Figure S2. Temporal dynamics of heterogeneity of tumor and adjacent normal tissue T cells, related to Figures 2 and 3.

(**A**) T cell subsets in adjacent cecum at 2, 4, and 6 weeks after AKP organoid implantation based on multiomic analysis. (**B**) Flow cytometric analysis of the frequencies of Helios$^+$ and RORγt$^+$ Treg subsets in indicated tissues at indicated time points after AKP tumor implantation. (**C**) Flow cytometric analysis of the frequency of "time-stamped" YFP$^+$ cells within total Treg population in indicated tissues on day 14 of tumor growth in *Foxp3*^{CreER}*Rosa26*$^{lsl\text{-}YFP}$ mice pre-treated with tamoxifen 9 and 6 days prior to AKP tumor organoid implantation. Sham, mice injected with vehicle. Injected, mice injected with tumor organoids. (**D–E**) Flow cytometric analysis of the frequency of IL-10$^+$(tdTomato$^+$) Treg cells within tagged YFP$^+$ Treg population (**D**) and the frequency of tagged YFP$^+$ cells within IL-10$^+$(tdTomato$^+$) Treg population (**E**) in indicated tissues one day before tumor implantation (D-1) or on day 14 of AKP tumor growth in *Il10*^{CreER}*Rosa26*$^{lsl\text{-}YFP}$*Foxp3*$^{Thy1.1}$ mice pre-treated with tamoxifen 9 and 6 days prior to tumor organoid implantation. For (**B–E**), data are pooled from two independent experiments and are mean ± s.e.m. Statistical tests were performed using unpaired *t*-tests.

Figure S3. TCR- and IL-2-dependent gene expression in tumoral IL-10$^+$ and IL-10$^-$ Treg cell subsets, related to Figures 2 and 3.

(**A**) chromVAR analysis of differential motif enrichment for all Treg subsets with the indicated tissue origins (T, tumor; C, cecum). (**B**) Violin plots of gene scores of genes associated with TCR-signaling (30 genes). (**C–D**) The expression of *Il17a*, *Il17f*, and *Il1rl1* (**C**) and *Pdcd1*, *Ctla4*, and *Havcr2* (**D**) in indicated AKP tumoral T cell subsets at indicated time points after tumor implantation.

Figure S4. Selective depletion of IL-10$^+$ and IL-10$^-$ Treg cells does not alter overall immune cell abundance or T cell subset composition of AKP tumors, related to Figures 4 and 5.

(**A–E**) Flow cytometric analysis of the frequencies of DTR$^+$ cell (**A**), the absolute number of Helios$^+$ Treg cell per milligram of tumor (**B**), the absolute number of RORγt$^+$ Treg cells per milligram of tumor (**C**), and the frequency of total CD45$^+$ (**D**), CD8$^+$ and CD4$^+$ Tconv cell (**E**) populations in DT and control (Ctrl) bDT-treated *Il10*^{Cre} *Foxp3*$^{lsl\text{-}DTR}$ (top) and *Il10*^{Cre} *Foxp3*$^{flox\text{-}DTR}$ mice (bottom). Data are pooled from two independent experiments and are shown as mean ± s.e.m. Statistical tests were performed using unpaired *t*-tests.

Figure S5. Selective IL-10$^+$ and IL-10$^-$ Treg loss results in distinct changes in myeloid cell and CD4$^+$ T cell compositions of the AKP tumor microenvironment, related to Figures 4 and 5.

(**A**) Flow cytometric analysis of the frequencies of monocytes, macrophages, neutrophils, mast cells, and eosinophils in AKP tumors from indicated treatment groups of *Il10*^{Cre} *Foxp3*$^{lsl\text{-}DTR}$ (top) and *Il10*^{Cre} *Foxp3*$^{flox\text{-}DTR}$ mice (bottom) two weeks after AKP organoid implantation. (**B**) *Il22* expression as measured by scRNA-seq analysis in indicated cell populations. (**C**) UMAP plots depicting non-T cell immune cells from IL10$^+$ Treg depleted (DT) and Ctrl tumors (n=5,028 cells; DT: 3,165 cells from 5 mice; Ctrl: 1,863 cells from 4 mice). (**D**) Stacked bar plots comparing the composition of non-T cell immune cells in Ctrl and DT conditions. (**E**) Volcano plots depicting Wilcoxon tests for indicated populations from DT vs Ctrl mice. For (**A**), data are pooled from two independent experiments and are mean ± s.e.m. Statistical analysis was performed using unpaired *t*-tests.

Figure S6. IL-17A promotes *In vitro* growth of AKP tumor cells, related to Figure 5.

Number of AKP tumor cells recovered after 7 days of culture with indicated cytokines or vehicle (Ctrl). Data are pooled from two independent experiments and are mean ± s.e.m. Statistical tests were performed using one-way ANOVA.

Figure S7. Single-cell analysis of human T cells and IL-10$^+$ and IL-10$^-$ Treg subsets in CRC patients, related to Figure 6.

(**A**) Proportions of T cell subsets grouped by tissue (left) and by patient (right). Cell types are annotated as in Figure 6B. (**B**) Expression of marker genes differentiating IL-10$^+$ and IL-10$^-$ Treg cells. (**C**) Dot plot of marker genes for T cell subsets. (**D**) UMAP of T cell subsets (left, n=18,198 cells) and tissue distribution (right) identified in published scRNA-seq data.[45] (**E**) Bar graphs depicting the number of cells for each cell type and the proportions for each tissue (colon/tumor). (**F**) Expression of *FOXP3* to highlight the Treg cells. (**G**) Marker genes of T cell subsets.

Figure S8. Cross dataset analysis of IL-10$^+$ and IL-10$^-$ Treg subsets in CRC and their association with survival probabilities, related to Figure 6.
(**A**) Violin plots of gene scores calculated on the cells from published scRNA-seq dataset[45] using indicated gene modules identified in Figure 3D. (**B**) Predictions of cell types in published spatial transcriptomic dataset. (**C**) Spatial plots highlighting the predictions of IL-10$^+$ and IL-10$^-$ Treg cells in tumor and NAT. (**D**) Bulk RNA-seq from CRC patients (n=102) deconvolved into cell types. Kaplan-Meier survival curves using quantile cutoffs on the prediction proportions of IL-10$^+$ (left) and IL-10$^-$ Treg cells (right).

Figure S9. Pan-cancer analysis of human IL-10$^+$ and IL-10$^-$ Treg subsets, related to Figure 6.
(**A**) UMAP visualization of previously identified Treg clusters from 14 tumor types, metastases, and adjacent healthy tissue from cancer patients and PBMC, bone marrow, and lymph nodes from healthy donors (n=30,031 cells).[46] (**B**) Gene scores of modules identified in Figure 6F. (**C**) Expressions of select genes distinguishing Treg clusters. (**D**) Composition of the Treg subsets across 14 cancer types. AML: Acute myeloid leukemia, BCC: Basal cell carcinoma, BRCA: Breast cancer, CRC: Colorectal cancer, DLBCL: Diffuse large B cell lymphoma, FL: Follicular lymphoma, GBM: Glioblastoma multiforme, HCC: Hepatocellular carcinoma, HNSC: Head and neck squamous cell carcinoma, LGG: Low-grade glioma, NSCLC: Non-small cell lung cancer, PDAC: Pancreatic ductal adenocarcinoma, SKCM: Skin cutaneous melanoma, STAD: Stomach adenocarcinoma.

Figure S10. Gating strategy for T cells and myeloid cells, related to STAR Methods.
(**A–B**) Example gating strategy for T cells (**A**) and myeloid cells (**B**).

Table S1. Characteristics of human patients, related to Figures 6 and S8.

Table S2. Differentially expressed genes and gene modules in Treg subsets, related to Figures 3, 6, S8, and S9.

Figure S1

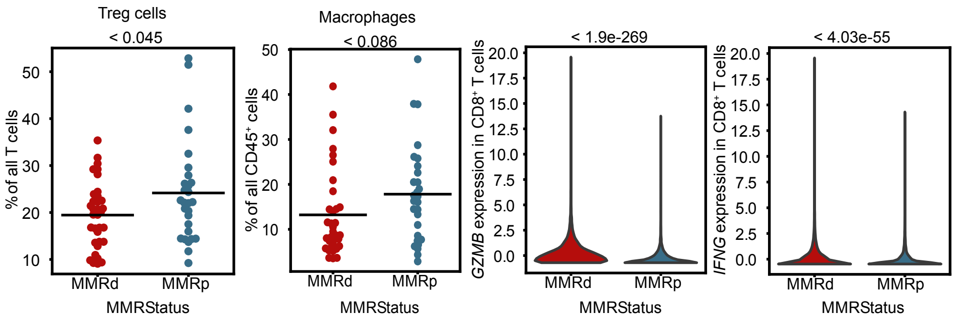

Treg cells
< 0.045
% of all T cells
MMRd
MMRp
MMRStatus
Macrophages
< 0.086
% of all CD45+ cells
MMRd
MMRp
MMRStatus
< 1.9e-269
GZMB expression in CD8+ T cells
MMRd
MMRp
MMRStatus
< 4.03e-55
IFNG expression in CD8+ T cells
MMRd
MMRp
MMRStatus

Figure S2

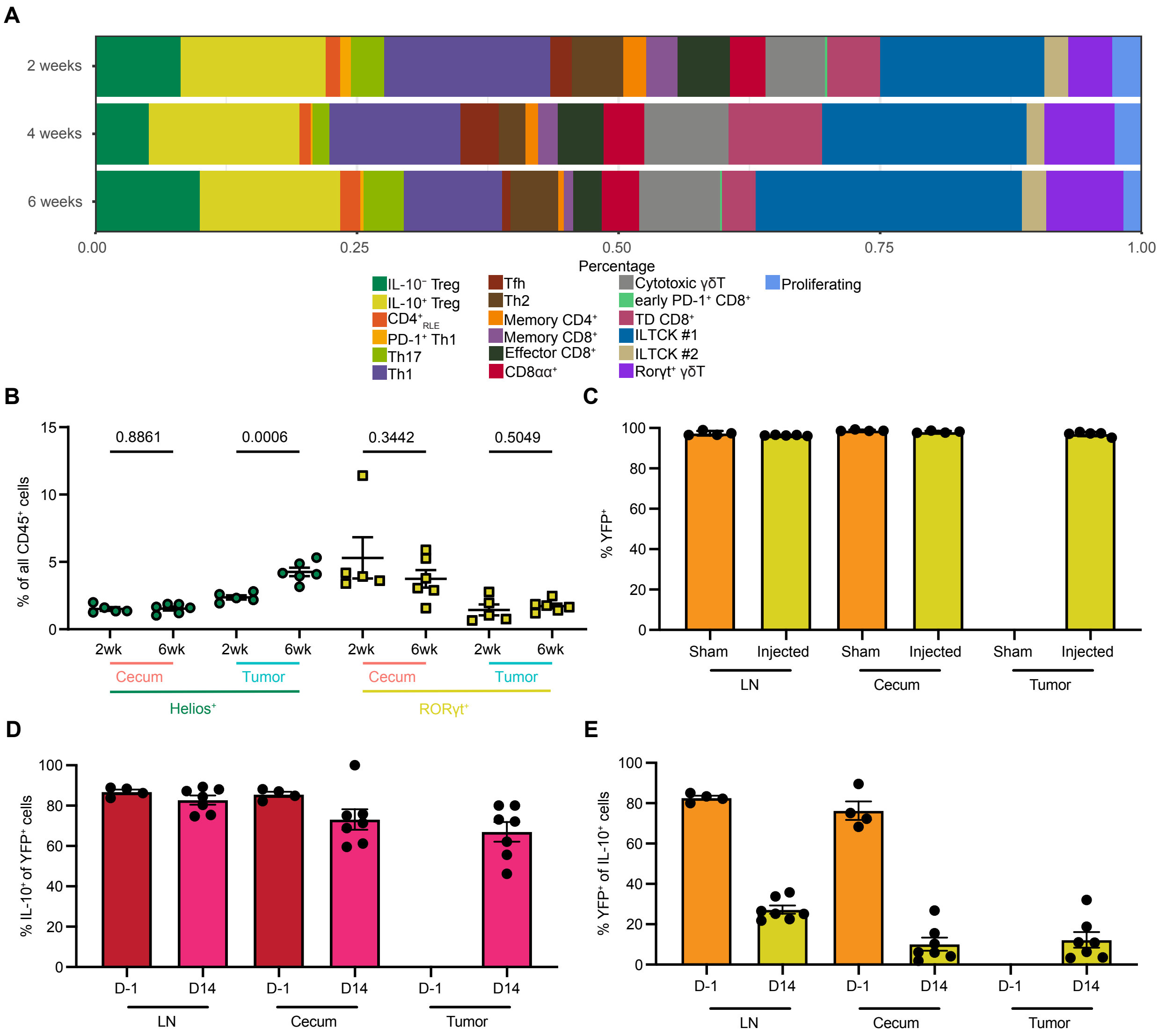

A
2 weeks
4 weeks
6 weeks
0.00
0.25
0.50
0.75
1.00
Percentage
IL-10⁻ Treg
IL-10⁺ Treg
CD4⁺RLE
PD-1⁺ Th1
Th17
Th1
Tfh
Th2
Memory CD4⁺
Memory CD8⁺
Effector CD8⁺
CD8αα⁺
Cytotoxic γδT
early PD-1⁺ CD8⁺
TD CD8⁺
ILTCK #1
ILTCK #2
Rorγt⁺ γδT
Proliferating
B
% of all CD45⁺ cells
0.8861
0.0006
0.3442
0.5049
2wk
6wk
Cecum
Tumor
Helios⁺
RORγt⁺
C
% YFP⁺
Sham
Injected
LN
Cecum
Tumor
D
% IL-10⁺ of YFP⁺ cells
D-1
D14
LN
Cecum
Tumor
E
% YFP⁺ of IL-10⁺ cells
D-1
D14
LN
Cecum
Tumor

Figure S3

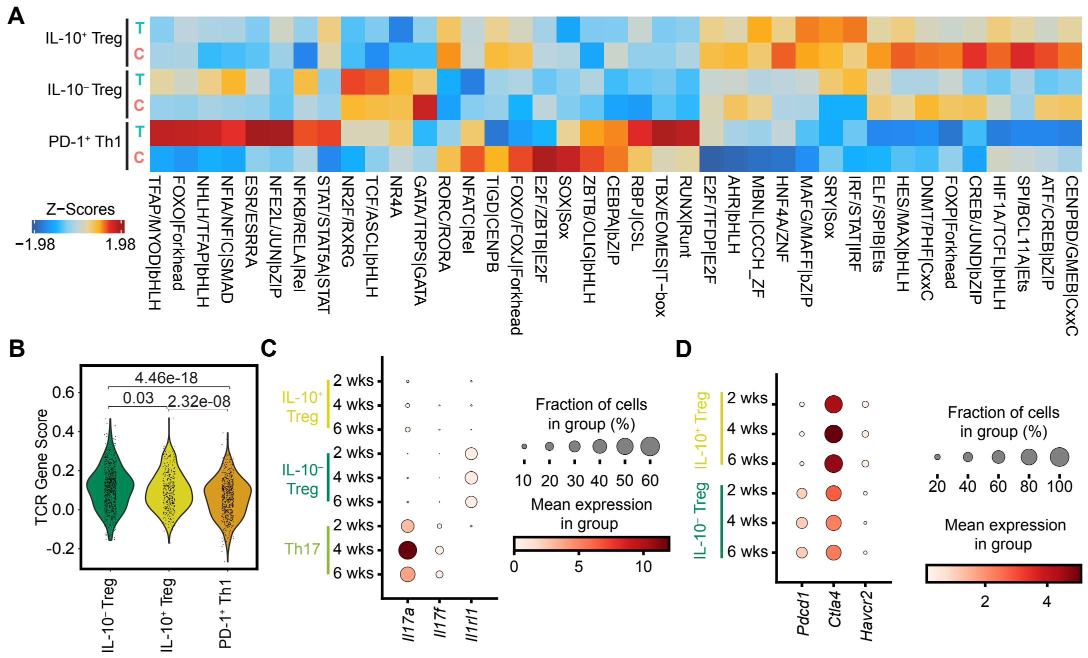
A
IL-10⁺ Treg
IL-10⁻ Treg
PD-1⁺ Th1
T
C
Z−Scores
−1.98
1.98
TFAP/MYOD|bHLH
FOXO|Forkhead
NHLH/TFAP|bHLH
NFIA/NFIC|SMAD
ESR/ESRRA
NFE2L/JUN|bZIP
NFKB/RELA|Rel
STAT/STAT5A|STAT
NR2F/RXRG
TCF/ASCL|bHLH
NR4A
GATA/TRPS|GATA
RORC/RORA
NFATC|Rel
TIGD|CENPB
FOXO/FOXJ|Forkhead
E2F/ZBTB|E2F
SOX|Sox
ZBTB/OLIG|bHLH
CEBPA|bZIP
RBPJ|CSL
TBX/EOMES|T−box
RUNX|Runt
E2F/TFDP|E2F
AHR|bHLH
MBNL|CCCH_ZF
HNF4A/ZNF
MAFG/MAFF|bZIP
SRY|Sox
IRF/STAT|IRF
ELF/SPIB|Ets
HES/MAX|bHLH
DNMT/PHF|CxxC
FOXP|Forkhead
CREB/JUND|bZIP
HIF1A/TCFL|bHLH
SPI/BCL11A|Ets
ATF/CREB|bZIP
CENPBD/GMEB|CxxC
B
TCR Gene Score
4.46e-18
0.03
2.32e-08
0.6
0.4
0.2
0.0
-0.2
IL-10⁻ Treg
IL-10⁺ Treg
PD-1⁺ Th1
C
IL-10⁺ Treg
IL-10⁻ Treg
Th17
2 wks
4 wks
6 wks
Il17a
Il17f
Il1rl1
Fraction of cells in group (%)
10 20 30 40 50 60
Mean expression in group
0 5 10
D
IL-10⁺ Treg
IL-10⁻ Treg
2 wks
4 wks
6 wks
Pdcd1
Ctla4
Havcr2
Fraction of cells in group (%)
20 40 60 80 100
Mean expression in group
2 4

Figure S4

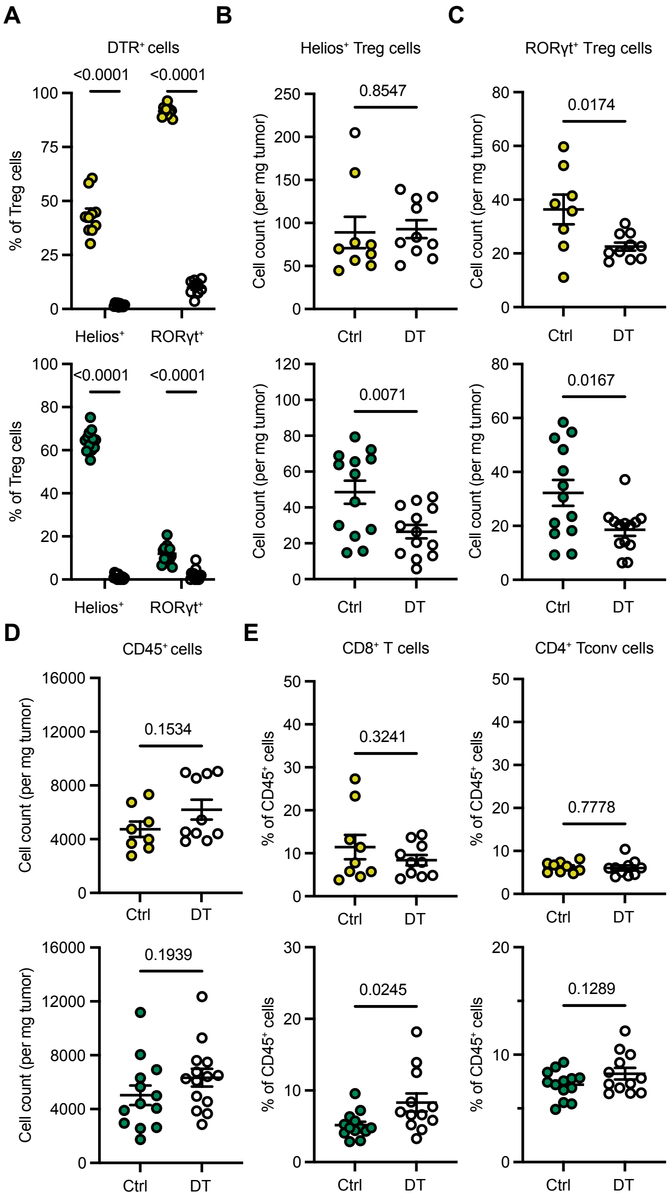
A
B
C
DTR+ cells
Helios+ Treg cells
RORγt+ Treg cells
<0.0001
<0.0001
0.8547
0.0174
% of Treg cells
Cell count (per mg tumor)
Helios+
RORγt+
Ctrl
DT
0.0071
0.0167
D
E
CD45+ cells
CD8+ T cells
CD4+ Tconv cells
0.1534
0.3241
0.7778
% of CD45+ cells
0.1939
0.0245
0.1289

Figure S5

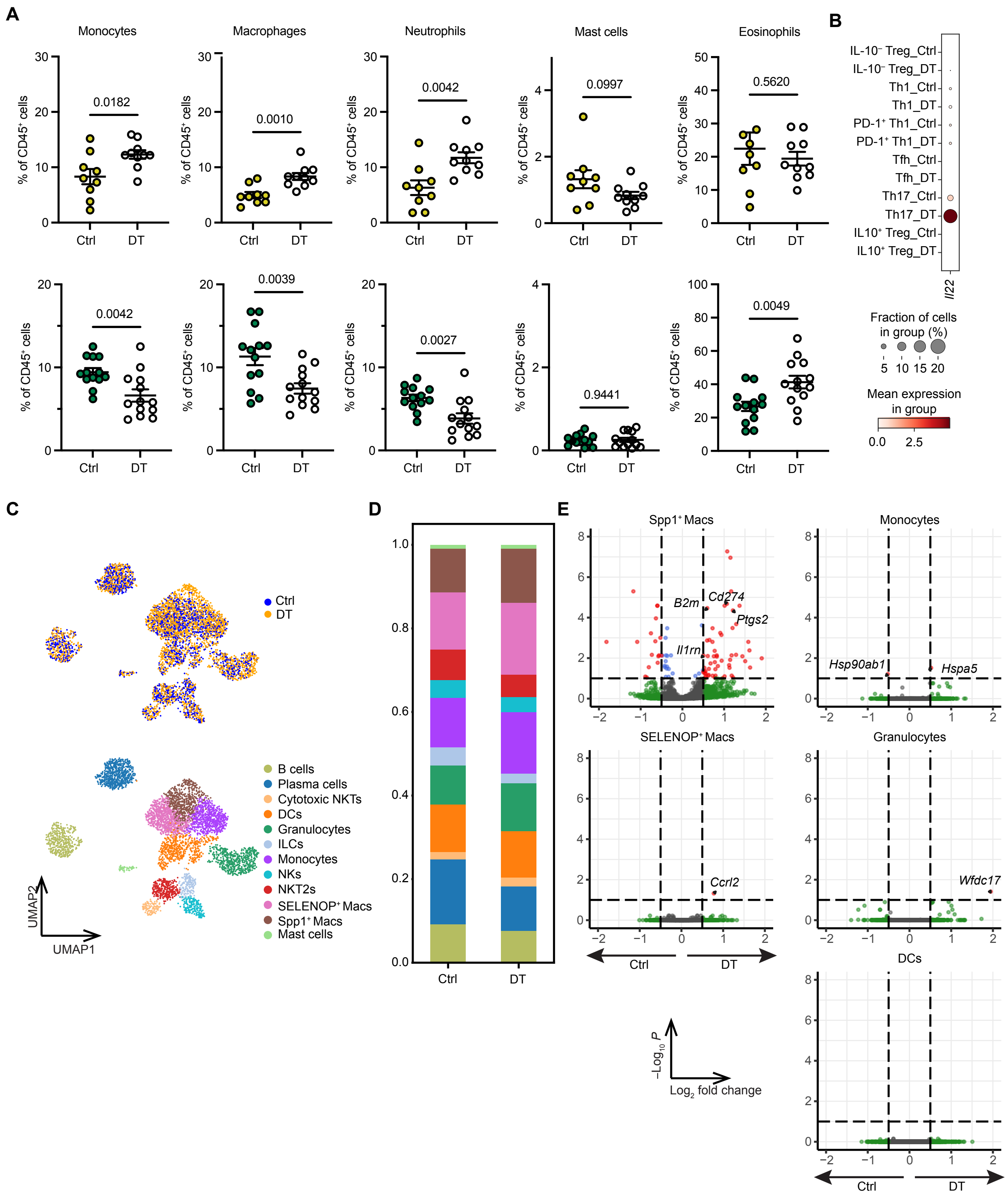
A
Monocytes
Macrophages
Neutrophils
Mast cells
Eosinophils
% of CD45⁺ cells
Ctrl
DT
0.0182
0.0010
0.0042
0.0997
0.5620
0.0042
0.0039
0.0027
0.9441
0.0049
B
IL-10⁻ Treg_Ctrl
IL-10⁻ Treg_DT
Th1_Ctrl
Th1_DT
PD-1⁺ Th1_Ctrl
PD-1⁺ Th1_DT
Tfh_Ctrl
Tfh_DT
Th17_Ctrl
Th17_DT
IL10⁺ Treg_Ctrl
IL10⁺ Treg_DT
Il22
Fraction of cells in group (%)
5 10 15 20
Mean expression in group
0.0 2.5
C
Ctrl
DT
B cells
Plasma cells
Cytotoxic NKTs
DCs
Granulocytes
ILCs
Monocytes
NKs
NKT2s
SELENOP⁺ Macs
Spp1⁺ Macs
Mast cells
UMAP2
UMAP1
D
E
Spp1⁺ Macs
B2m
Cd274
Ptgs2
Il1rn
Monocytes
Hsp90ab1
Hspa5
SELENOP⁺ Macs
Ccrl2
Granulocytes
Wfdc17
DCs
−Log10 P
Log2 fold change

Figure S6

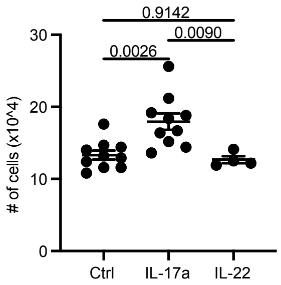

Figure S7

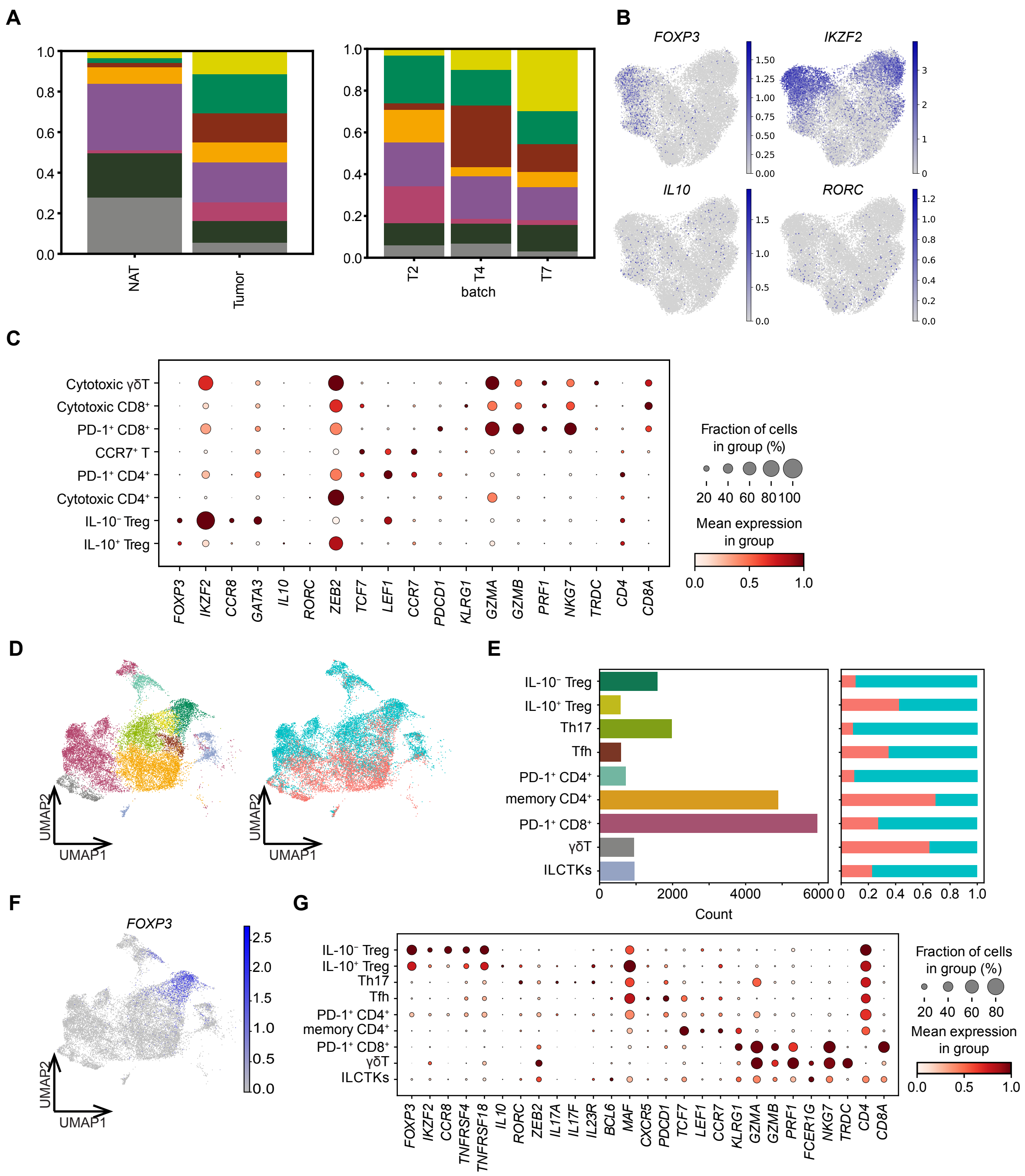

A
NAT
Tumor
T2
T4
T7
batch
B
FOXP3
IKZF2
IL10
RORC
C
Cytotoxic γδT
Cytotoxic CD8+
PD-1+ CD8+
CCR7+ T
PD-1+ CD4+
Cytotoxic CD4+
IL-10- Treg
IL-10+ Treg
Fraction of cells in group (%)
Mean expression in group
D
UMAP1
UMAP2
E
IL-10- Treg
IL-10+ Treg
Th17
Tfh
PD-1+ CD4+
memory CD4+
PD-1+ CD8+
γδT
ILCTKs
Count
F
FOXP3
G
Fraction of cells in group (%)
Mean expression in group

Figure S8

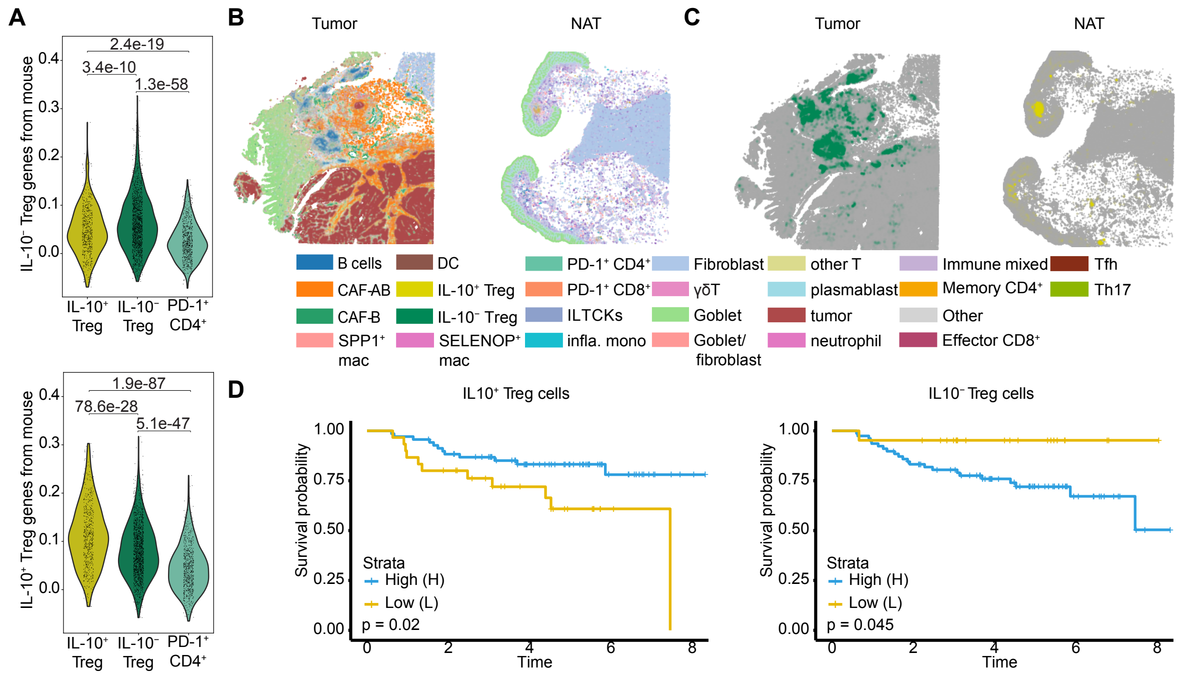
A
B
C
D
2.4e-19
3.4e-10
1.3e-58
IL-10⁻ Treg genes from mouse
IL-10⁺ Treg
IL-10⁻ Treg
PD-1⁺ CD4⁺
1.9e-87
78.6e-28
5.1e-47
IL-10⁺ Treg genes from mouse
Tumor
NAT
B cells
DC
PD-1⁺ CD4⁺
Fibroblast
other T
Immune mixed
Tfh
CAF-AB
IL-10⁺ Treg
PD-1⁺ CD8⁺
γδT
plasmablast
Memory CD4⁺
Th17
CAF-B
IL-10⁻ Treg
ILTCKs
Goblet
tumor
Other
SPP1⁺ mac
SELENOP⁺ mac
infla. mono
Goblet/ fibroblast
neutrophil
Effector CD8⁺
IL10⁺ Treg cells
IL10⁻ Treg cells
Survival probability
Time
Strata
High (H)
Low (L)
p = 0.02
p = 0.045

Figure S9

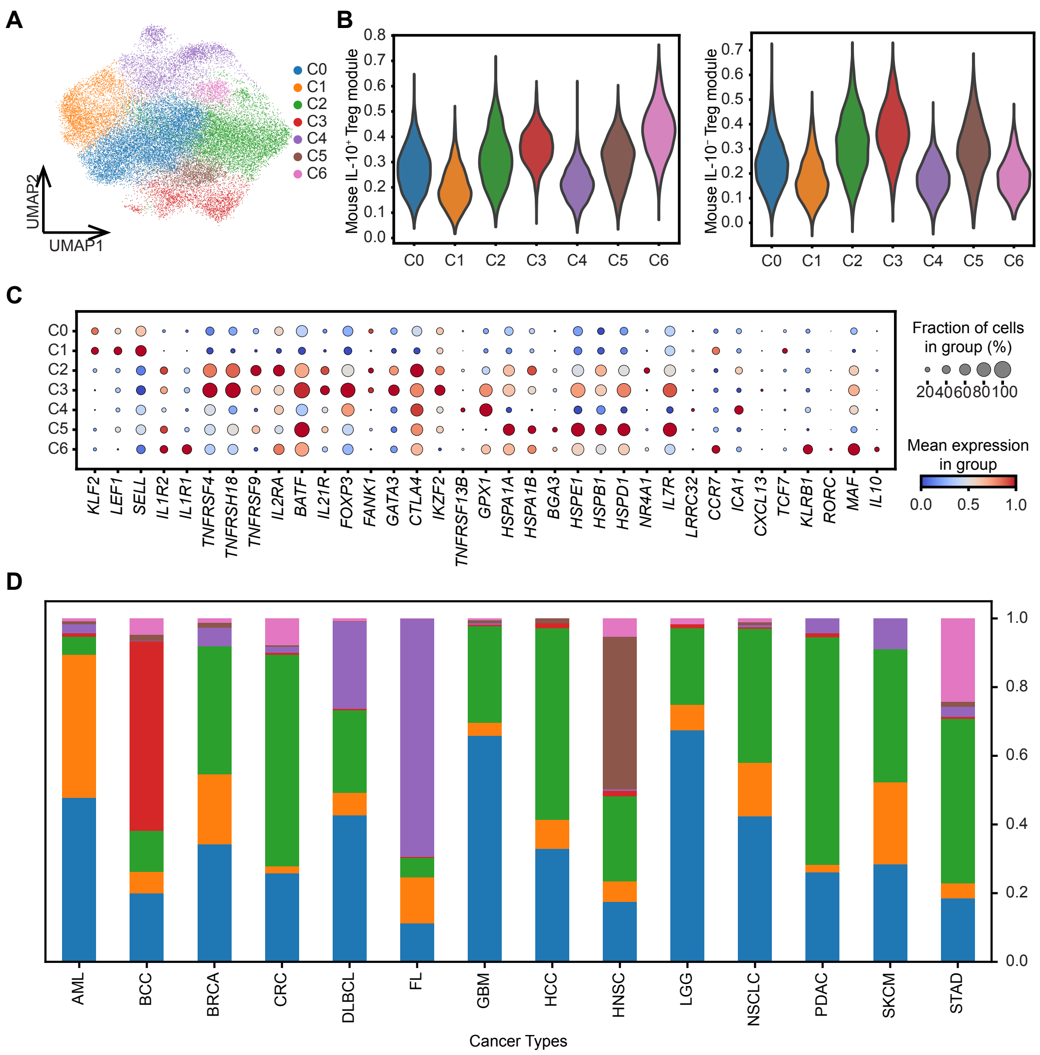

A
UMAP2
UMAP1
C0
C1
C2
C3
C4
C5
C6
B
Mouse IL-10⁺ Treg module
Mouse IL-10⁻ Treg module
C
Fraction of cells in group (%)
20 40 60 80 100
Mean expression in group
0.0 0.5 1.0
KLF2 LEF1 SELL IL1R2 IL1R1 TNFRSF4 TNFRSH18 TNFRSF9 IL2RA BATF IL21R FOXP3 FANK1 GATA3 CTLA4 IKZF2 TNFRSF13B GPX1 HSPA1A HSPA1B BGA3 HSPE1 HSPB1 HSPD1 NR4A1 IL7R LRRC32 CCR7 ICA1 CXCL13 TCF7 KLRB1 RORC MAF IL10
D
AML BCC BRCA CRC DLBCL FL GBM HCC HNSC LGG NSCLC PDAC SKCM STAD
Cancer Types

Figure S10

A **Gating strategy for T cells**

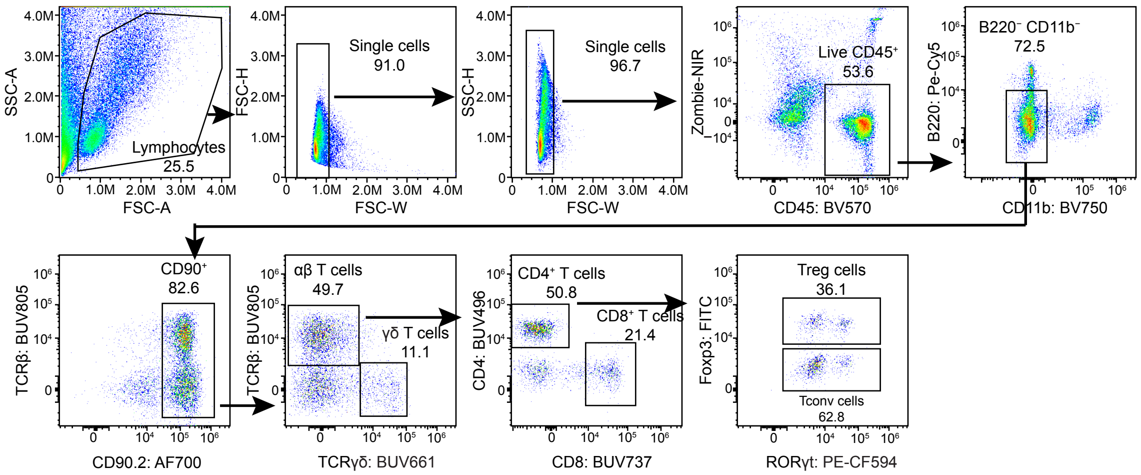

B **Gating strategy for myeloid cells**

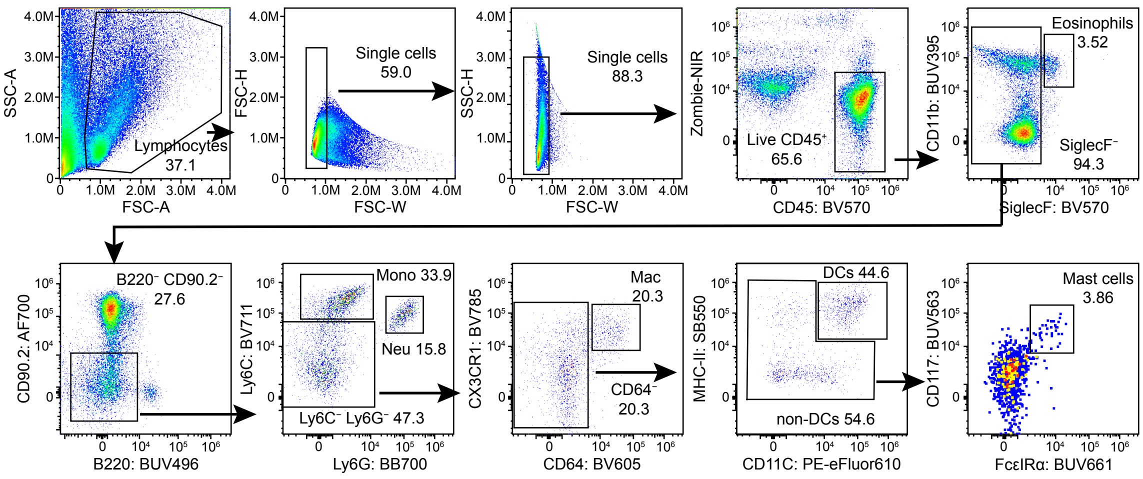

Table S1

SampleID	gender	Age@Dx	Ethnicity(NOS There is evidence, other than surname or maiden name, that the person is Hispanic, but he/she cannot be assigned to any of the other categories 1-5.)	RACE (A - asian, AI - asian/indian,BAA-black african american, R - refused,HPI-Hawaiian/PacificIslander, W-WHITE)	Diagnosis_Date(BiopsyDate)	MS status	Experimental Analysis
SCMP2	F	64	Non-Hispanic	W	7/9/24	MSS	Single cell multiomic analysis
SCMP4	M	63	Non-Hispanic	W	12/18/23	MSS	Single cell multiomic analysis
SCMP7	M	49	Non-Hispanic	W	8/8/24	MSS	Single cell multiomic analysis
OPR001	F	73	Non-Hispanic	W	4/9/14	Unknown	Bulk RNA-seq
OPR010	F	52	Non-Hispanic	W	10/23/14	Unknown	Bulk RNA-seq
OPR013	F	68	Non-Hispanic	W	10/27/14	Unknown	Bulk RNA-seq
OPR024	M	66	Hispanic or Latino	W	6/24/15	Unknown	Bulk RNA-seq
OPR027	F	50	Non-Hispanic	W	9/3/15	Unknown	Bulk RNA-seq
OPR029	M	66	Non-Hispanic	W	10/14/15	Unknown	Bulk RNA-seq
OPR030	M	44	Non-Hispanic	W	10/19/15	Unknown	Bulk RNA-seq
OPR032	M	67	Non-Hispanic	W	10/30/15	Unknown	Bulk RNA-seq
OPR036	F	64	Non-Hispanic	BAA	3/18/16	Unknown	Bulk RNA-seq
OPR039	M	41	Hispanic or Latino	W	4/8/16	Unknown	Bulk RNA-seq
OPR041	M	57	Non-Hispanic	W	6/3/16	Unknown	Bulk RNA-seq
OPR042	M	61	Non-Hispanic	BAA	6/8/16	Unknown	Bulk RNA-seq
OPR044	M	51	Non-Hispanic	W	7/14/16	Unknown	Bulk RNA-seq
OPR048	F	71	Non-Hispanic	W	8/19/16	Unknown	Bulk RNA-seq
OPR050	M	64	Non-Hispanic	W	12/16/16	Unknown	Bulk RNA-seq
OPR051	F	47	Non-Hispanic	W	1/10/17	Unknown	Bulk RNA-seq
OPR052	M	62	Non-Hispanic	W	1/12/17	Unknown	Bulk RNA-seq
OPR057	F	70	Non-Hispanic	W	6/8/17	Unknown	Bulk RNA-seq
OPR060	M	52	Non-Hispanic	W	7/17/17	Unknown	Bulk RNA-seq
OPR061	M	51	Non-Hispanic	W	7/17/17	Unknown	Bulk RNA-seq
OPR062	F	51	Non-Hispanic	W	7/31/17	Unknown	Bulk RNA-seq
OPR064	M	71	Non-Hispanic	A	8/10/17	Unknown	Bulk RNA-seq
OPR070	M	56	Non-Hispanic	W	2/2/18	Unknown	Bulk RNA-seq
OPR072	M	68	Non-Hispanic	W	5/23/18	Unknown	Bulk RNA-seq
OPR078	M	74	Non-Hispanic	W	9/17/18	Unknown	Bulk RNA-seq
JM209	M	50	Non-Hispanic	W	5/3/17	Unknown	Bulk RNA-seq
JM210	M	58	Non-Hispanic	W	5/18/17	Unknown	Bulk RNA-seq
JM212	M	63	Non-Hispanic	W	6/21/17	Unknown	Bulk RNA-seq
JM213 (CILARC)	F	54	Non-Hispanic	W	6/14/17	Unknown	Bulk RNA-seq
JM214	M	54	Non-Hispanic	W	6/19/017	Unknown	Bulk RNA-seq
JM215	M	47	Hispanic	W	5/30/17	Unknown	Bulk RNA-seq
JM217	M	33	Non-Hispanic	A	7/7/17	Unknown	Bulk RNA-seq
JM219	M	35	Non-Hispanic	W	3/15/13	Unknown	Bulk RNA-seq
JM220	F	45	Non-Hispanic	W	3/7/14	Unknown	Bulk RNA-seq
JM221	M	87	Non-Hispanic	W	3/10/14	Unknown	Bulk RNA-seq
JM222	M	66	Non-Hispanic	W	4/1/14	Unknown	Bulk RNA-seq
JM223	M	38	Non-Hispanic	W	4/10/14	Unknown	Bulk RNA-seq
JM224	M	37	Non-Hispanic	W	5/9/14	Unknown	Bulk RNA-seq
JM226	M	45	Non-Hispanic	AI	9/19/14	Unknown	Bulk RNA-seq
JM229	M	60	Non-Hispanic	Refused	9/23/14	Unknown	Bulk RNA-seq
JM231	F	63	Non-Hispanic	W	7/7/15	Unknown	Bulk RNA-seq
JM232	F	51	Non-Hispanic	W	9/25/15	Unknown	Bulk RNA-seq
JM234	F	45	Non-Hispanic	W	11/20/15	Unknown	Bulk RNA-seq
JM235	F	55	Non-Hispanic	W	12/16/15	Unknown	Bulk RNA-seq
JM236	M	59	Non-Hispanic	W	12/18/15	Unknown	Bulk RNA-seq
JM237	M	66	Non-Hispanic	W	1/22/16	Unknown	Bulk RNA-seq
JM238	M	53	Non-Hispanic	W	12/23/16	Unknown	Bulk RNA-seq
JM240	F	46	Non-Hispanic	A	1/12/17	Unknown	Bulk RNA-seq
JM241	M	54	Non-Hispanic	W	1/23/17	Unknown	Bulk RNA-seq
JM242	F	61	Non-Hispanic	W	1/23/17	Unknown	Bulk RNA-seq
JM243 (CILARC)	F	58	Non-Hispanic	W	2/13/17	MSS	Bulk RNA-seq
JM244	F	51	Non-Hispanic	W	2/15/17	Unknown	Bulk RNA-seq
JM245	M	51	Non-Hispanic	Other	2/15/17	Unknown	Bulk RNA-seq
JM246	M	52	Non-Hispanic	W	4/7/17	Unknown	Bulk RNA-seq
JM247	F	66	Non-Hispanic	W	4/10/17	Unknown	Bulk RNA-seq
JM248	M	55	Non-Hispanic	AI	4/1/17	Unknown	Bulk RNA-seq
JM249	M	52	Non-Hispanic	W	6/16/17	Unknown	Bulk RNA-seq
JM250 (CILARC)	F	86	Non-Hispanic	W	6/16/17	MSS	Bulk RNA-seq
JM252	F	44	Non-Hispanic	W	6/29/17	Unknown	Bulk RNA-seq
JM253 (CILARC)	M	40	Non-Hispanic	W	6/26/17	MSS	Bulk RNA-seq
JM256	M	56	Non-Hispanic	W	9/13/17	Unknown	Bulk RNA-seq
JM258	F	52	Non-Hispanic	W	9/14/17	Unknown	Bulk RNA-seq

JM259 (CILARC)	M	56	Non-Hispanic	W	9/28/17	MSS	Bulk RNA-seq
JM262	M	57	Non-Hispanic	W	11/2/17	Unknown	Bulk RNA-seq
JM264	M	85	Non-Hispanic	W	9/26/17	Unknown	Bulk RNA-seq
JM265	F	45	Non-Hispanic	W	11/27/17	Unknown	Bulk RNA-seq
JM268	M	46	Non-Hispanic	W	1/4/18	Unknown	Bulk RNA-seq
JM269	M	65	Non-Hispanic	W	2/5/18	Unknown	Bulk RNA-seq
JM270	M	49	Hispanic	W	3/19/18	MSS	Bulk RNA-seq
JM271	F	47	Non-Hispanic	W	3/20/18	Unknown	Bulk RNA-seq
JM272 (CILARC)	M	42	Non-Hispanic	BAA	3/23/18	Unknown	Bulk RNA-seq
JM274	M	48	Hispanic	W	4/9/18	Unknown	Bulk RNA-seq
JM275	M	44	Non-Hispanic	W	4/16/18	Unknown	Bulk RNA-seq
JM279 (CILARC)	M	70	Non-Hispanic	W	5/16/18	MSS	Bulk RNA-seq
JM280 (CILARC)	M	45	Non-Hispanic	W	5/17/18	MSS	Bulk RNA-seq
JM281 (CILARC)	M	45	Non-Hispanic	Refused	1/5/18	MSS	Bulk RNA-seq
JM284	M	58	Non-Hispanic	W	3/12/19	Unknown	Bulk RNA-seq
JM288 (CILARC)	M	44	Non-Hispanic	W	6/19/18	MSS	Bulk RNA-seq
JM289	M	65	Hispanic	W	7/6/18	Unknown	Bulk RNA-seq
JM290 (CILARC)	F	30	Non-Hispanic	W	7/6/18	MSS	Bulk RNA-seq
JM291	F	50	Non-Hispanic	W	7/6/18	Unknown	Bulk RNA-seq
JM299 (CILARC)	F	52	n-Spanish; Non-Hispa	W	8/13/18	MSS	Bulk RNA-seq
JM304	M	65	Spanish NOS	W	10/1/18	Unknown	Bulk RNA-seq
JM305 (CILARC)	M	53	n-Spanish; Non-Hispa	W	12/3/18	MSS	Bulk RNA-seq
JM306	F	70	n-Spanish; Non-Hispa	W	11/28/18	Unknown	Bulk RNA-seq
JM310 (CILARC)	M	51	n-Spanish; Non-Hispa	W	1/4/19	MSS	Bulk RNA-seq
JM313	M	70	Unknown	W	1/21/19	Unknown	Bulk RNA-seq
JM314 (CILARC)	F	66	n-Spanish; Non-Hispa	W	1/17/19	MSS	Bulk RNA-seq
JM316 (CILARC)	M	68	n-Spanish; Non-Hispa	W	1/23/19	MSS	Bulk RNA-seq
JM320 (CILARC)	F	29	n-Spanish; Non-Hispa	W	2/22/19	MSI	Bulk RNA-seq
JM321 (CILARC)	F	51	n-Spanish; Non-Hispa	W	2/12/19	MSS	Bulk RNA-seq
JM322 (CILARC)	F	47	n-Spanish; Non-Hispa	BAA	2/15/19	MSS	Bulk RNA-seq
JM324 (CILARC)	M	63	n-Spanish; Non-Hispa	W	3/5/19	MSS	Bulk RNA-seq
JM325 (CILARC)	M	62	Spanish NOS	W	3/7/19	MSS	Bulk RNA-seq
JM328 (CILARC)	M	39	Spanish NOS	W	3/5/19	MSI	Bulk RNA-seq
JM329	M	61	n-Spanish; Non-Hispa	W	4/3/19	Unknown	Bulk RNA-seq
JM334	M	64	n-Spanish; Non-Hispa	W	4/23/19	Unknown	Bulk RNA-seq
JM337 (CILARC)	F	49	n-Spanish; Non-Hispa	AI	5/1/19	MSS	Bulk RNA-seq
JM338 (CILARC)	M	43	Non-Hispanic	W	5/21/19	MSS	Bulk RNA-seq
JM343	F	70	Non-Hispanic	W	8/5/19	Unknown	Bulk RNA-seq
JM346	M	62	Non-Hispanic	W	9/5/19	Unknown	Bulk RNA-seq
JM349 (CILARC)	M	47	Non-Hispanic	A	9/11/19	MSS	Bulk RNA-seq

Table S2

Mouse		Human	
**Il10- Tregs**	**Il10+ Tregs**	**IL10- Tregs**	**IL10+ Tregs**
Ikzf2	Il10	IKZF2	NAB1
Il1rl1	Maf	GK	RORA
Gata3	Ctla4	FILIP1L	PTPRC
Rel	Ccr2	GK-AS1	FYN
Tox	Nckap5	RTKN2	CD247
Flnb	Ece1	CADM1	MBNL1
Tnfrsf9	Zeb2	EPSTI1	IL2RA
Nfkb1	Itm2b	CMSS1	TRAF3
Tent5a	Ikzf3	AC105402.3	SYTL3
Tmtc2	Rabgap1l	PLCL1	CCR6
Epas1	Icos	VAV3	SIK3
Rora	Havcr2	MALT1	NEK7
Il18rap	Gzmb	AL136456.1	UTY
Klrg1	Lag3	AC093865.1	GNG2
Cd83	Gm36975	TIAM1	PLEKHG1
Mgat5	Neb	CRADD	PDE4B
Dgat2	Gas2	TNFRSF9	ZC3H12D
Swap70	Cytip	AC104365.1	USP9Y
Myo1e	Galnt2	RDX	HIVEP1
Ikzf4	Ccr5	FNDC3B	MAP3K5
Tspan13	Il1r1	THADA	TAB2
Dusp10	Pde4b	FANK1	FTH1
Pdcd1	Gcnt2	USP15	SEC14L1
Gm28112	Olfr60	STAM	ARAP2
Stat4	Lrrfip2	TBC1D4	NAMPT
Pcgf5’	4930503L19Rik	ZC3H12C	SRGN
Pkp4	E130308A19Rik	FAM184A	GLCCI1
Zbtb46	Gm13481	AC104850.2	BICDL1
Ly75	Ccr1	LINC02099	RORA-AS1
Itgb8	Atp10a	OSBPL6	CNOT6L
Ttn	Snx9	CACNB2	MYO5A
Pcyt1a	Gm49890	ICA1	PRKCH
Gda	Agfg1	PICALM	CD44
Il2ra	Ppp1r14c	GATA3	JAZF1
Uap1	Tiparp	SNED1	ADAM12
Zc3h12c	Il23r	ETS1	JAK1
Il18r1	Matn2	MAGEH1	CRYBG1
Epb41l2	Rgs1	NR3C1	ZMYM2
Traf3	Fmnl2	IL1RL1	G3BP2
Cep85l	Phactr2	AP000462.1	LDLRAD4
Rbpj	Farp1	ATP13A3	USP47
Stat1	Irak3	AL137009.1	RAPGEF6
Itgav	Eea1	NIBAN1	CERK
Ankrd33b	Ubash3b	NCAM1	DUSP16
Bcl2l1	St6galnac3	SORL1	RNF19A
Fam129a	Lamc1	LAYN	CDC42SE2
Ttc39c	Got1	REL	ZNF292
Ap3b1	Smap2	CEP120	TNIK
Ppp1r16b	Wdfy2	NCOA2	KAT2B
Myo10	Rbm24	SMCHD1	HPGD
Apaf1	Ston2	FAAH2	RNF149
Itga4	Adam12	IQGAP2	PPP2R5C
Rnf157	Twsg1	GPCPD1	JMJD1C
Nrip1	Hnrnpll	STK17B	AKAP13
	Ets2	TMTC2	EVL
	Timp2	ZNF331	STAT4
	Dock10	ZEB1	RASGRP1
	Ier5l	TNFRSF11A	FNBP1
	Arl4c	FLNB	SATB1
		ITPKB	CBLB
		MIR3142HG	PLCG2
		PDCD4	PRKY
		RAP1GDS1	RFTN1
		BATF	ZNF831

AP001011.1	USP48
CCR8	GPR183
CAST	PHACTR2
SLAMF1	KMT2E
CD55	CAMK4
MAML3	DDX3Y
BACH1	APBB1IP
FAM13A	GPR15
PMAIP1	IL7R
IL12RB2	IKZF1
LEF1	RFX3
DGKH	PRDM2
NCALD	TRPS1
TSIX	AC016831.7
MYO1E	SSH1
GPHN	TOX
GADD45A	ICOS
CDK14	GSPT1
LINC00519	CREM
DGKE	HDAC4
CLNK	SAMSN1
ID3	GNB1
SGPP1	FOXO1
BARD1	UXS1
SLC9A7	RALGAPA1
PHLPP1	ZHX2
DUSP4	AC006460.1
CD27	MIR4435-2HG
HERC5	PBX4
MAP4K3	USP12
TRIM25	EML4
TIGIT	MBD2
ITGB1	TUT7
CPEB4	BRAF
ACSL4	LINC00513
MAGI1	DTHD1
BTG1	PCBP3
HPRT1	BTD
PCED1B	MAST4
FCRL3	IL1R1
CREB3L2	AGPAT4
SELL	MGAT4A
GRK3	ZEB2
TSPAN13	MAP3K4
CARD16	PTPN13
SIPA1L1	IL26
DNAH8	PTPRM
MBOAT7	RNF144B
FUT8	MACC1
	CYTH1
	ANKRD28
	FOXP1
	LY75
	GTDC1
	RAPGEF1
	MAF
	IKZF3
	RBPJ
	RBMS1
	VPS13C
	ADAM19
	ADTRP
	MGAT5
	BRD9
	AC104078.2
	AC083837.1
	LRP12
	HES4

SERINC5
AL390957.1
IL17A
SAMD12
GPRIN3
P2RY14
RGS6
SYTL2
PKIG
PPARG
ZNRF1
ZZEF1
ATP2B4
LINC02384
ABCA1
THEMIS
RAP1B
CELF2
SOX5
IL23R
PKHD1
CXCL8
MIR181A1HG
CD6
PLAGL1
AUTS2
IFNG-AS1
CD2
FURIN
IL1R2
HIVEP2
NFKB1
OSTF1
ARHGEF7
ANTXR2
CTSH
CCR4
NOTCH2
PHF21A
ABTB2
TGFBR2
COL5A3
GRAMD1B
PTPN4
KLRB1
LAG3
C2CD4A
CDK6
BLM
C1GALT1
IL22
SUSD4
ST8SIA4
PTPN2
AC017002.5
MTRNR2L1
AC023590.1
AAK1
LGR4
GZMA
SERINC3
CD3D
STARD4
AC007271.1
LINC00299
CYTH3
VMP1
LYPLAL1-AS1
